# A discontinuous Poisson–Boltzmann equation with interfacial jump: homogenisation and residual error estimate

**DOI:** 10.1080/00036811.2015.1105962

**Published:** 2015-11-04

**Authors:** Klemens Fellner, Victor A. Kovtunenko

**Affiliations:** a Institute of Mathematics and Scientific Computing, University of Graz, NAWI Graz, 8010Graz, Austria.; b Lavrent’ev Institute of Hydrodynamics, 630090Novosibirsk, Russia.

**Keywords:** Electro-kinetic, steady-state Poisson–Nernst–Planck system, Boltzmann statistics, nonlinear Poisson equation, Robin condition, interfacial jump, oscillating coefficients, homogenisation, error corrector, 35B27, 35J60, 78A57, 82B24

## Abstract

A nonlinear Poisson–Boltzmann equation with inhomogeneous Robin type boundary conditions at the interface between two materials is investigated. The model describes the electrostatic potential generated by a vector of ion concentrations in a periodic multiphase medium with dilute solid particles. The key issue stems from interfacial jumps, which necessitate discontinuous solutions to the problem. Based on variational techniques, we derive the homogenisation of the discontinuous problem and establish a rigorous residual error estimate up to the first-order correction.

## 1. Introduction

In this paper, we consider the steady-state problem of a nonlinear Poisson–Nernst–Planck (PNP) system, which describes multiple concentrations of charged particles (e.g. ions) subject to a self-consistent electrostatic potential calculated from Poisson’s equation. In particular, we shall investigate the PNP model on a multiphase medium. The prototypical multiphase medium in mind consists of an electrolyte medium (pores), which surrounds disjoint solid particles. Such models have numerous applications describing electro-kinetic phenomena in bio-molecular or electro-chemical models, photo-voltaic systems and semiconductors, see e.g. [1–6] and references therein. Our specific interests are motivated by models of Li-Ion batteries, see the relevant references [7–10].

We shall deal with the nonlinearity of the model within an analytic framework, in which the PNP system can be transformed into an equivalent scalar Poisson–Boltzmann (PB) equation, see e.g. [11–13]. This is possible, when reaction terms in the charged particle fluxes are omitted and the equations for the concentrations decouple since the charged particle concentrations are explicitly determined by the corresponding Boltzmann statistics. At the interface boundaries, this implies homogeneous Neumann conditions, which nevertheless allow for jumps in the concentrations of the species. For references applying linearisation of the PNP equations near the Boltzmann distribution see [14,15].

In order to be able to homogenise the equations over the entire pores-and-particles medium, a physically consistent continuation of the governing relations in the particles is needed. Here, we assume the Gouy–Chapman-Stern model for electric double layers (EDLs).[16] By this, charged species underlie transport through diffusion and electrostatic drift in the pores and pure diffusion in the solid phase. This model proposes a jump of the electrostatic field across the interface (a voltage drop) and a current prescribed at the interior boundary of the solid particles.

In our general setting, we assume that the current may have a jump across the interface as well. Allowing for jumps extends the modelling to describe e.g. a separator in super-capacitors.

In the following, we will derive a discontinuous formulation of the PB equation (valid both on the volume occupied by the solid particles and on the surrounding porous space). The key feature addressed in this manuscript is the imposed inhomogeneous jump conditions, which complement the PB equation (see (8) below).

We emphasise that, from the point of view of partial differential equations, the considered PB equations are characterised by a strongly nonlinear term, which is unbounded and features exponential growth, see e.g. [17].

A first aim of this paper is to establish a proper variational setting of the problem, while a second part deals with its rigorous homogenisation. With respect to the later, the averaged effective coefficients of the limit problem represent the macroscopic behaviour of the EDL, which is of primary practical importance.

For reference concerning the classic homogenisation theories, we refer to [18–23]. The applied methods range from two-scale convergence (see e.g. [24]) over Gamma-convergence (see e.g. [25]) to unfolding (see [26]) and others. While formal methods of averaging are widely used in the literature, their verification in terms of residual error estimates is a hard task.

From the point of view of homogenisation, the principal difficulty of discontinuous problems concerns the nonstandard boundary conditions with jumps: firstly, related jump conditions are inherent for cracks. For models and methods used in crack problems, we refer to [27–29] and references therein. From a geometric viewpoint, cracks are open manifolds in the reference domain. Hence, classic Poincare–Friedrichs–Korn inequalities are valid in such situations. In contrast to cracks, the interfaces here are assumed to be closed manifolds disconnecting the reference domain. This fact requires discontinuous versions of Poincare–Friedrichs–Korn inequalities, which are then applied for semi-norm estimates.

Secondly, the interface boundary conditions are of Robin type. The homogenisation results known for linear problems with Robin (also called Fourier) conditions are crucially sensitive to the asymptotic rates of the involved homogenisation parameters. This issue concerns the coefficients in the boundary condition (cf. Lemma 1 below) and the volume fraction of solid particles in periodic cells (cf. Lemma2 below), see e.g. [24,30–32].

In the literature, on the one hand, the fully coupled PNP as well as decoupled PB equations are treated mostly under homogeneous boundary conditions of the Neumann type, see [11,12,14]. On the other hand, even inhomogeneous Neumann and also Robin boundary conditions are homogenised for the most part of linear equations, see e.g. [26,31]. In e.g. [4,6] only the pore model is described and extended in the solid phase inexplicitly.

Homogenisation of transmission problems with interface jumps can be found in works concerning models of diffusion in a partially fractured porous medium, models of heat conduction in composite materials or in models of absorption of a dissolved chemical in a fluid flowing through a porous medium. We refer to [33–36] for linear transmission problems as well as for linear problems under nonlinear [37] and contact [38] boundary conditions, and to [39] for a semi-linear case with bounded nonlinearity.

In comparison to the works mentioned above, the principal challenge of the underlying, discontinuous PB equation is the strongly nonlinear term together with nonstandard jump conditions of Robin type, which allow not only discontinuous fields but also discontinuous fluxes across a separator. In the present contribution, we homogenise it and derive the averaged limit problem. A further major result is a rigorous estimate of the residual error up to the first-order correction.

For these purposes, we develop a variational technique based on orthogonal Helmholtz decomposition following the lines of [21,23]. In a periodic cell, we decompose oscillating coefficients (describing the electric permittivity) by using the nontrivial kernel in the space of vector valued periodic functions, which is represented by sums of constant and divergence free (and thus, skew symmetric) vector fields (cf. Lemma 3). Employing solutions of appropriately defined discontinuous cell problems, we obtain a regular decomposition of the homogenisation problem (see Theorem 2).

A second result establishes the critical rates of the asymptotic behaviour with respect to a homogenisation parameter $\varepsilon ↘0^{+}$ for coefficients in the inhomogeneous transmission condition: we find on the one side that the critical rate for the coefficient by interfacial jumps is $\frac{1}{\varepsilon }$. This factor occurs in the discontinuous Poincare inequality (for the norm squared, cf. (21) below) and is thus relevant for a coercivity estimate, which in return contributes to the solvability of the discontinuous problem and the subsequent estimate of the homogenisation error.

On the other side, the critical rate for the flux prescribed at the interior boundary of solid particles is ε. At this rate, the interior boundary flux induces an additional potential, which distributes over the macroscopic domain in the homogenisation limit $\varepsilon ↘0^{+}$. If the asymptotic rate is lower than the critical one, then this flux vanishes in the limit. Otherwise, if the asymptotic rate is bigger, then the flux term diverges.

From the above description, we summarise the key points of this paper as follows:

- the study of inhomogeneous interfacial conditions describing EDL;
- the combination of the strongly nonlinear term of exponential type, jumps and Robin conditions;
- a variational framework of the discontinuous problem;
- the performing of the homogenisation procedure with rigorous error estimates; and
- the identification of the critical asymptotic rates of the boundary coefficients.

**Outline:** In Sections 2.1–2.3, we first present the problem geometry, the physical and the mathematical model. Section 2.3 establishes moreover the equivalence of the steady state of the PNP system with the scalar Poisson–Boltzmann (PB) equation and the existence of a unique solution to the PB equation (see Theorem 1).

In Section 3, we consider the homogenisation problem and the residual error estimate. At first, we state three auxiliary Lemmata before stating the main homogenisation Theorem 2.

Finally, Section 4 provides a brief discussion of the obtained results.

## 2. Statment of the Problem

We start with the description of the geometry.

### 2.1. Geometry

Let ω denote the domain (which is an open set) occupied by solid particles of general shape (either single or multiple particles), which are located inside the unit cell $\Upsilon ={\left(0,1\right)}^{d}\subset R^{d}$, d=1,2,3. We assume that all particles ω⊂Υ are disjunctively located as well as bounded away from the boundary ∂Υ, i.e. ω∩∂Υ=∅.

**Figure 1.**
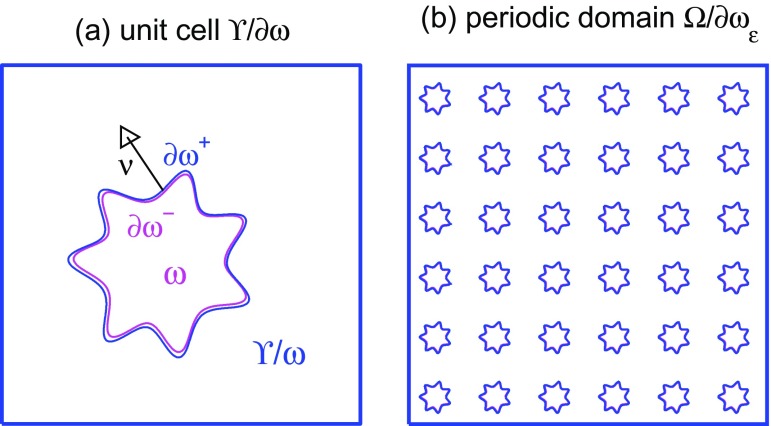
Two-dimensional example geometry with one star-shaped particle: (a) the unit cell and (b) the periodic disjoint domains $\Omega \backslash \partial \omega_{\varepsilon }$.

We assume that the boundary ∂ω is Lipschitz continuous with outer normal vector $\nu ={\left(\nu_{1},\cdots ,\nu_{d}\right)}^{⊤}$ pointing away from the domain ω. Moreover, we distinguish the positive (outward orientated) surface $\partial \omega^{+}$ and the negative (inward orientated) surface $\partial \omega^{-}$ as the faces of the boundary ∂ω, when approaching the boundary ∂ω from outside, i.e. from $\Upsilon \backslash \bar{\omega }$ or from the inside, i.e. from ω, respectively. For a two-dimensional example configuration see the illustration in Figure 1(a).

In the following, we consider a fixed, small homogenisation parameter $\varepsilon \in R_{+}$ and pave $R^{d}$ with periodic cells $\Upsilon_{p}^{\varepsilon }$ indexed by p∈N. The periodic cells $\Upsilon_{p}^{\varepsilon }$ are constructed from Υ in the following way: The position of every spatial point $x={\left(x_{1},\cdots ,x_{d}\right)}^{⊤}\in R^{d}$ can be decomposed as

$$x=\varepsilon \left\lfloor \frac{x}{\varepsilon }\right\rfloor +\varepsilon \left\{\frac{x}{\varepsilon }\right\},\;\;\left\lfloor \frac{x}{\varepsilon }\right\rfloor \in Z^{d},\;\left\{\frac{x}{\varepsilon }\right\}\in \Upsilon ,$$

into the integer-valued floor function coordinates $\left\lfloor \frac{x}{\varepsilon }\right\rfloor \in Z^{d}$ and the fractional coordinates $\{\frac{x}{\varepsilon }\}\in \Upsilon$. We shall then enumerate all possible integer vectors $\left\lfloor \frac{x}{\varepsilon }\right\rfloor$ by means of a natural ordering with the index p∈N. According to this index, we associate $\varepsilon \lfloor \frac{x}{\varepsilon }\rfloor$ with the pth cell $\Upsilon_{p}^{\varepsilon }$ and $\varepsilon \{\frac{x}{\varepsilon }\}=\varepsilon y$ shall denote the local coordinates in all cells which correspond to y∈Υ.

We will denote by $\omega_{p}^{\varepsilon }\subset \Upsilon_{p}^{\varepsilon }$ the respective solid particles obtained by means of the paving with $\{\frac{x}{\varepsilon }\}=y$ for y∈ω. We note that the rescaling does not change the unit outer normal vector ν.

Evidently, the periodic mapping x↦y, $R^{d}\mapsto \Upsilon$, is surjective. This construction can be generalised to an arbitrary orthotope Υ, see [26].

Let Ω be the reference domain in $R^{d}$ with Lipschitz boundary ∂Ω and denote again the outer normal vector by ν. By reordering the index p, it is then possible to account for all solid particles $\omega_{p}^{\varepsilon }\subset \Omega$ with the index set $p=1,\cdots ,N_{\varepsilon }$, see [26,40]. We remark that $N_{\varepsilon }=O\left(\varepsilon^{-d}\right)$ as $\varepsilon ↘0^{+}$.

By omitting solid particles which are ‘too close’ to the external boundary ∂Ω, we shall ensure a constant gap with the distance O(ε) between ∂Ω and all particles $\omega_{p}^{\varepsilon }$. Thus, Ω is divided into the multiple domains $\omega_{\varepsilon }:=\cup_{p=1}^{N_{\varepsilon }}\omega_{p}^{\varepsilon }$ corresponding to all the solid particles located periodically in the reference domain and the remaining porous space $\Omega \backslash {\bar{\omega }}_{\varepsilon }$.

In the following, we shall denote by $\partial \omega_{\varepsilon }=\cup_{p=1}^{N_{\varepsilon }}\partial \omega_{p}^{\varepsilon }$ the union of boundaries $\partial \omega_{p}^{\varepsilon }$ and introduce the multiply-connected domains

$$\Omega \backslash \partial \omega_{\varepsilon }=\left(\Omega \backslash {\bar{\omega }}_{\varepsilon }\right)\cup \omega_{\varepsilon },\;\omega_{\varepsilon }:=\cup_{p=1}^{N_{\varepsilon }}\omega_{p}^{\varepsilon },\;\partial \omega_{\varepsilon }=\cup_{p=1}^{N_{\varepsilon }}\partial \omega_{p}^{\varepsilon }.$$

Moreover, for functions ξ, which are discontinuous over the interface $\partial \omega_{\varepsilon }$, we will denote the jump across the interface by

$$\left[\;\left[\xi \right]\;\right]:=\xi^{+}-\xi^{-},\;\xi^{\pm }{:=\xi |}_{\partial \omega_{\varepsilon }^{\pm }}.$$

Here, $\partial \omega_{\varepsilon }^{+}=\cup_{p=1}^{N_{\varepsilon }}{\left(\partial \omega_{p}^{\varepsilon }\right)}^{+}$ summarises the positive faces (orientated towards the interior of the pore space $\Omega \backslash {\bar{\omega }}_{\varepsilon }$), and $\partial \omega_{\varepsilon }^{-}=\cup_{p=1}^{N_{\varepsilon }}{\left(\partial \omega_{p}^{\varepsilon }\right)}^{-}$ accounts for the negative faces (orientated towards the interior of the solid phase $\omega_{\varepsilon }$).

### 2.2. Physical model

In the heterogeneous domain $\Omega \backslash \partial \omega_{\varepsilon }$, which consist of the particle volumes $\omega_{\varepsilon }$ and the porous space $\Omega \backslash {\bar{\omega }}_{\varepsilon }$, we consider the electrostatic potential ϕ and (n+1) components of concentrations of charged particles $c={\left(c_{0},\cdots ,c_{n}\right)}^{⊤}$, n≥1. The physical consistency requires positive concentrations c>0.

At the external boundary ∂Ω, we shall impose Dirichlet boundary conditions $\phi =\phi^{\mathrm{bath}}$ and $c=c^{\mathrm{bath}}$ corresponding to a surrounding bath and given by constant values $\phi^{\mathrm{bath}}\in R$ and $c^{\mathrm{bath}}={\left(c_{0}^{\mathrm{bath}},\cdots ,c_{n}^{\mathrm{bath}}\right)}^{⊤}\in R_{+}^{n+1}$. We can then consider the normalised electrostatic potential $\phi -\phi^{\mathrm{bath}}$ and concentrations $c/c^{\mathrm{bath}}$ (i.e. $c_{s}/{c_{s}}^{\mathrm{bath}}$ for all s=0,⋯,n) and prescribe the following normalised Dirichlet conditions:

(1) $$\begin{array}{c} \phi =0,\;c=1\;\;\text{on}\partial \Omega . \end{array}$$

In the following, all further relations will be formulated for the normalised potential and concentrations such that (1) holds.

Let $z_{s}\in R$ denote the electric charge of the sth species with concentration $c_{s}$ for s=0,⋯,n. For the n+1-components of charges particles, we shall assume the following charge-neutrality

(2) $$\begin{array}{c} \sum_{s=0}^{n}z_{s}c_{s}{|}_{\partial \Omega }=\sum_{s=0}^{n}z_{s}=0 \end{array}$$

because of the normalisation supposed in (1). A necessary condition for (2) is $\min_{s\in \{0,\cdots ,n\}}z_{s}<0<\max_{s\in \{0,\cdots ,n\}}z_{s}$.

The charge-neutrality is used to justify the Boltzmann statistics when extending the PNP equations to the solid phase with Ohm’s law, see equations (5) and Proposition 2 below.

Moreover, the charge-neutrality assumption (2) implies also the following strong monotonicity property

(3) $$\begin{array}{c} {K|\xi |}^{2}\leq -\sum_{s=0}^{n}z_{s}\xi \exp\left(-z_{s}\xi \right)\;\;\text{for all}\xi \in R\;\left(K>0\right), \end{array}$$

for a constant K>0, which follows directly from Taylor expansion with respect to $\left(-z_{s}\xi \right)$, see [11, Lemma 15].

We consider the following PNP steady-state system consisting of (n+2) nonlinear, homogeneous equations:

(4a) $$\begin{array}{cc} -\mathrm{div}(D_{s}\nabla c_{s}) & =0,\;s=0,\cdots ,n,\;\;\text{in}\omega_{\varepsilon }, \end{array}$$

(4b) $$\begin{array}{cc} -\mathrm{div}(D_{s}\left(\nabla c_{s}+\frac{z_{s}}{\kappa T}c_{s}\nabla \phi \right)) & =0,\;s=0,\cdots ,n,\;\;\text{in}\Omega \backslash {\bar{\omega }}_{\varepsilon }, \end{array}$$

(5a) $$\begin{array}{cc} -\mathrm{div}(A^{\varepsilon }\nabla \phi ) & =0,\;\;\text{in}\omega_{\varepsilon }, \end{array}$$

(5b) $$\begin{array}{cc} -\mathrm{div}\left(A^{\varepsilon }\nabla \phi \right)-\sum_{s=0}^{n}z_{s}c_{s} & =0,\;\;\text{in}\Omega \backslash {\bar{\omega }}_{\varepsilon }. \end{array}$$

In both equations (4), $D_{s}\in L^{\infty }{\left(\Omega \right)}^{d\times d}$, $D_{s}>0$, s=0,⋯,n denote symmetric and positive definite diffusion matrices, which are in general discontinuous over $\partial \omega_{\varepsilon }$. In (4b), κ>0 is the Boltzmann constant, and T>0 is the temperature. We remark that the form of (4b) is based on assuming the Einstein relations for the mobilities. Moreover, equation (4a) models the effect of charges particles being included into the solid particles, which is well known, for instance, for $Li^{+}$-ions, see e.g. [16].

In (5), $A\in L^{\infty }{\left(\Upsilon \right)}^{d\times d}$ denotes the symmetric and positive definite matrix of the electric permittivity, which oscillates periodically over cells according to $A^{\varepsilon }\left(x\right):=A\left(\left\{\frac{x}{\varepsilon }\right\}\right)$ and satisfies

(6) $$\begin{array}{cc} & A^{⊤}\left(y\right)=A\left(y\right),\;y\in \Upsilon \\ & \underline{K}{\left|\xi \right|}^{2}\leq \xi^{⊤}A\left(y\right)\xi \leq \bar{K}{\left|\xi \right|}^{2}\;\forall \xi \in R^{d},y\in \Upsilon ,\;\left(0<\underline{K}<\bar{K}\right). \end{array}$$

The entries of the permittivity matrix A are discontinuous functions in the cell Υ across the interface ∂ω. A typical example considers piecewise constant $A=\sigma_{\omega }I$ in ω and $A=\sigma_{\Upsilon }I$ in $\Upsilon \backslash \bar{\omega }$, with material parameters $\sigma_{\omega }>0$ and $\sigma_{\Upsilon }>0$, where I denotes here the identity matrix in $R^{d\times d}$. In the following, we denote by $A_{ij}$, i,j=1,⋯,d, the matrix entries of A.

From a physical point of view, (5a) represents Ohm’s law in the solid phase. Moreover, we remark that the equations on $\omega_{\varepsilon }$, i.e. (4a) for c and (5a) for ϕ are linear while the equations (4b) and (5b) on $\Omega \backslash {\bar{\omega }}_{\varepsilon }$ form a coupled nonlinear problem on the porous space.

The modelling of boundary conditions at the interfaces is a delicate issue. For the charge carries fluxes in (4), we assume homogeneous Neumann conditions

(7a) $$\begin{array}{cc} {\left(\nabla c_{s}^{-}\right)}^{⊤}D_{s}\nu & =0,\;s=0,\cdots ,n,\;\;\text{on}\;\partial \omega_{\varepsilon }^{-}, \end{array}$$

(7b) $$\begin{array}{cc} {\left(\nabla c_{s}^{+}+\frac{z_{s}}{\kappa T}c_{s}^{+}\nabla \phi^{+}\right)}^{⊤}D_{s}\nu & =0,\;s=0,\cdots ,n,\;\;\text{on}\;\partial \omega_{\varepsilon }^{+}. \end{array}$$

In fact, imposing inhomogeneous conditions in (7) will lead to quasi-Fermi statistics instead of the Boltzmann statistics, see (16) below. In particular inter-species reaction terms would pose significant difficulties, which are out of the scope of the present work.

For the electrostatic potential in (5), we suppose the following inhomogeneous interfacial boundary conditions (see the related physics in [16]):

(8a) $$\begin{array}{cc} {\left(\nabla \phi^{⊤}A^{\varepsilon }\right)}^{-}\nu -\frac{\alpha }{\varepsilon }\left[\;\left[\phi \right]\;\right] & =\varepsilon g,\;\;\;\text{on}\;\partial \omega_{\varepsilon }^{-}, \end{array}$$

(8b) $$\begin{array}{cc} -{\left(\nabla \phi^{⊤}A^{\varepsilon }\right)}^{+}\nu +\frac{\alpha }{\varepsilon }\left[\;\left[\phi \right]\;\right] & =0,\;\;\text{on}\;\partial \omega_{\varepsilon }^{+}. \end{array}$$

Here $\alpha \in R_{+}$ and g∈R are material parameters given at the interface. We note that by summing (8a) and (8b), we derive the relation

(9) $$\begin{array}{c} -\left[\;\left[\nabla \phi^{⊤}A^{\varepsilon }\right]\;\right]\nu =\varepsilon g,\;\;\text{on}\;\partial \omega_{\varepsilon }, \end{array}$$

implying that not only the electric potential ϕ but also fluxes $\nabla \phi^{⊤}A^{\varepsilon }\nu$ are discontinuous functions across the interface $\partial \omega_{\varepsilon }$ for g≠0.

The asymptotic weights $\frac{1}{\varepsilon }$ in front of [[ϕ]] and εg at the right-hand side of (8), which were already mentioned in the introduction, shall be discussed in detail during the below asymptotic analysis as $\varepsilon ↘0^{+}$.

We emphasise that the interface conditions (8) couple the porous phase $\Omega \backslash {\bar{\omega }}_{\varepsilon }$ with the solid phase $\omega_{\varepsilon }$ by means of the jump in [[ϕ]]. In fact, the jump conditions (8) can be compared with the following two cases of simplified boundary conditions: First, if ϕ were continuous across $\partial \omega_{\varepsilon }$, i.e. [[ϕ]]=0, then (8a) and (8b) would be decoupled into two usual Neumann boundary condition which do not represent the EDL. Second, if $\phi^{-}$ were known on the solid phase boundary $\partial \omega_{\varepsilon }^{-}$, then the model would be reduced to a model on the porous space $\Omega \backslash {\bar{\omega }}_{\varepsilon }$ with the following inhomogeneous Robin (called also Fourier) boundary condition (see [13])

$$\begin{array}{c} -{\left(\nabla \phi^{⊤}A^{\varepsilon }\right)}^{+}\nu +\frac{\alpha }{\varepsilon }\phi^{+}=\frac{\alpha }{\varepsilon }\phi^{-},\;\;\text{on}\partial \omega_{\varepsilon }^{+}. \end{array}$$

However, the subsequent homogenisation of this alternative model on the porous space $\Omega \backslash {\bar{\omega }}_{\varepsilon }$ would nevertheless require a suitable continuation of $\phi^{+}$ onto $\omega_{\varepsilon }$.

### 2.3. Mathematical model

In the following, we shall amend the state variables with the superscript ε in order to highlight the dependency on the cell size.

The physical model will be described by the following weak variational formulation of the boundary value problem (1), (4), (5), (7), (8): find an electrostatic potential $\phi^{\varepsilon }\in H^{1}\left(\Omega \backslash \partial \omega_{\varepsilon }\right)$ and n+1 components of charge carrier concentrations $c^{\varepsilon }\in H^{1}{\left(\Omega \backslash \partial \omega_{\varepsilon }\right)}^{n+1}\cap L^{\infty }{\left(\Omega \backslash \partial \omega_{\varepsilon }\right)}^{n+1}$ such that the concentrations are positive $c^{\varepsilon }>0$ and satisfy

(10) $$\begin{array}{cc} & \phi^{\varepsilon }=0,\;c^{\varepsilon }=1\;\;\text{on}\partial \Omega , \end{array}$$

(11) $$\begin{array}{cc} & \int_{\Omega \backslash \partial \omega_{\varepsilon }}(\nabla c_{s}^{\varepsilon }+\chi_{{}_{\Omega \backslash \omega_{\varepsilon }}}\frac{z_{s}}{\kappa T}\;c_{s}^{\varepsilon }\;\nabla \phi^{\varepsilon }{)}^{⊤}D_{s}\nabla c_{s}\;dx=0,\;s=0,\cdots ,n,\\ & \;\;\;\;\text{for all test-functions}\;c\in H^{1}{\left(\Omega \backslash \partial \omega_{\varepsilon }\right)}^{n+1}:c=0\;on\;\partial \Omega , \end{array}$$

(12) $$\begin{array}{cc} & \int_{\Omega \backslash \partial \omega_{\varepsilon }}\;\;\;({\left(\nabla \phi^{\varepsilon }\right)}^{⊤}A^{\varepsilon }\nabla \phi -\chi_{{}_{\Omega \backslash \omega_{\varepsilon }}}\sum_{s=0}^{n}z_{s}c_{s}^{\varepsilon }\phi )\;dx+\int_{\partial \omega_{\varepsilon }}\;\;\;\frac{\alpha }{\varepsilon }\left[\;\left[\phi^{\varepsilon }\right]\;\right]\left[\;\left[\phi \right]\;\right]\;dS_{x}\\ & \;\;\;=\int_{\partial \omega_{\varepsilon }^{-}}\varepsilon g\phi^{-}\;dS_{x}\;\;\;\text{for all}\phi \in H^{1}\left(\Omega \backslash \partial \omega_{\varepsilon }\right)\text{:}\phi =0\;\text{on}\partial \Omega . \end{array}$$

Here $\chi_{{}_{\Omega \backslash \omega_{\varepsilon }}}$ denotes the characteristic function of the set $\Omega \backslash \omega_{\varepsilon }$, and $H^{1}\left(\Omega \backslash \partial \omega_{\varepsilon }\right)$ is the usual Sobolev $H^{1}$-space defined on the multiply-connected domain $\Omega \backslash \partial \omega_{\varepsilon }=\left(\Omega \backslash {\bar{\omega }}_{\varepsilon }\right)\cup \omega_{\varepsilon }$ and endowed with the standard norm:

$${\left\|\phi \right\|}_{H^{1}\left(\Omega \backslash \partial \omega_{\varepsilon }\right)}^{2}={\left\|\phi \right\|}_{L^{2}\left(\Omega \backslash \partial \omega_{\varepsilon }\right)}^{2}+{\left\|\nabla \phi \right\|}_{L^{2}\left(\Omega \backslash \partial \omega_{\varepsilon }\right)}^{2}={\left\|\phi \right\|}_{H^{1}\left(\Omega \backslash \omega_{\varepsilon }\right)}^{2}+{\left\|\phi \right\|}_{H^{1}\left(\omega_{\varepsilon }\right)}^{2}.$$

Proposition 1: For strong solutions $\left(\phi^{\varepsilon },c^{\varepsilon }\right)$, the variational system (10)–(12) and the boundary value problem (1), (4), (5), (7), (8) are equivalent.

Proof: The assertion can be verified by usual variational arguments, which we briefly sketch for the sake of the reader. The variational equations (11) and (12) are derived by multiplying the equations (4), (5) with test-functions and subsequent integration by parts over $\Omega \backslash {\bar{\omega }}_{\varepsilon }$ and $\omega_{\varepsilon }$ due to boundary conditions (1) and (7), (8).

In return, given strong solutions $\left(\phi^{\varepsilon },c^{\varepsilon }\right)$, the boundary value problem (4), (5), (7), (8) is obtained by varying the test-functions (ϕ,c) in (11), (12) and with the help of the following Green’s formulas: by recalling the ν denotes both the outer normal on ∂Ω and ∂ω, we have for all $p\in L_{\mathrm{div}}^{2}{\left(\Omega \backslash \partial \omega_{\varepsilon }\right)}^{d}$

(13a) $$\begin{array}{cc} \int_{\Omega \backslash \omega_{\varepsilon }}\;\;\;p^{⊤}\nabla v\;dx & =-\int_{\Omega \backslash \omega_{\varepsilon }}\;\;\;v\;\mathrm{div}\left(p\right)\;dx-\int_{\partial \omega_{\varepsilon }^{+}}p^{⊤}v\nu \;dS_{x}\\ & \;+\int_{\partial \Omega }p^{⊤}v\nu \;dS_{x},\;\forall \;v\in H^{1}\left(\Omega \backslash \omega_{\varepsilon }\right), \end{array}$$

(13b) $$\begin{array}{cc} \int_{\omega_{\varepsilon }}\;\;\;p^{⊤}\nabla v\;dx & =-\int_{\omega_{\varepsilon }}\;\;\;v\;\mathrm{div}\left(p\right)\;dx+\int_{\partial \omega_{\varepsilon }^{-}}p^{⊤}v\nu \;dS_{x},\;\forall v\in H^{1}\left(\omega_{\varepsilon }\right), \end{array}$$

which are valid on $\Omega \backslash {\bar{\omega }}_{\varepsilon }$ and $\omega_{\varepsilon }$, respectively. Hence, by suming (13a) and (13b), we obtain the Green’s formula representation

(14) $$\begin{array}{c} \int_{\Omega \backslash \partial \omega_{\varepsilon }}\;\;\;p^{⊤}\nabla v\;dx=-\int_{\Omega \backslash \partial \omega_{\varepsilon }}\;\;\;v\;\mathrm{div}\left(p\right)\;dx-\int_{\partial \omega_{\varepsilon }}\left[\;\left[p^{⊤}v\right]\;\right]\nu \;dS_{x}+\int_{\partial \Omega }p^{⊤}v\nu \;dS_{x}, \end{array}$$

which holds on the disjoint domain $\Omega \backslash \partial \omega_{\varepsilon }$ for all $p\in L_{\mathrm{div}}^{2}{\left(\Omega \backslash \partial \omega_{\varepsilon }\right)}^{d}$ and $v\in H^{1}\left(\Omega \backslash \partial \omega_{\varepsilon }\right)$, see e.g. [29]. □

The following Proposition 2 states the crucial observation that introducing Boltzmann statistics allows to decouple the system of the homogeneous equations (11) and derive an equivalent scalar PB equation.

Proposition 2: The system (10)–(12) it is equivalent to the following nonlinear Poisson–Boltzmann equation: find $\phi^{\varepsilon }\in H^{1}\left(\Omega \backslash \partial \omega_{\varepsilon }\right)$ such that

(15a) $$\begin{array}{cc} & \phi^{\varepsilon }=0\;\;\text{on}\partial \Omega , \end{array}$$

(15b) $$\begin{array}{cc} & \int_{\Omega \backslash \partial \omega_{\varepsilon }}({\left(\nabla \phi^{\varepsilon }\right)}^{⊤}A^{\varepsilon }\nabla \phi -\sum_{s=0}^{n}z_{s}e^{-\frac{z_{s}}{\kappa T}\;\chi {}_{\Omega \backslash \omega_{\varepsilon }}\phi^{\varepsilon }}\phi )\;dx+\int_{\partial \omega_{\varepsilon }}\;\;\;\frac{\alpha }{\varepsilon }\left[\;\left[\phi^{\varepsilon }\right]\;\right]\left[\;\left[\phi \right]\;\right]\;dS_{x}=\int_{\partial \omega_{\varepsilon }^{-}}\varepsilon g\phi^{-}\;dS_{x}\\ & \;\;\;\text{for all test-functions}\;\phi \in H^{1}\left(\Omega \backslash \partial \omega_{\varepsilon }\right):\phi =0\;on\;\partial \Omega , \end{array}$$

together with the Boltzmann statistics determining $c^{\varepsilon }$ from $\phi^{\varepsilon }$, i.e.

(16) $$\begin{array}{cc} c_{s}^{\varepsilon } & =\exp(-\frac{z_{s}}{\kappa T}\phi^{\varepsilon }),\;s=0,\cdots ,n,\;\;\text{a.e. on}\Omega \backslash {\bar{\omega }}_{\varepsilon },\\ c_{s}^{\varepsilon } & \in R_{+},\;\;\;\;s=0,\cdots ,n,\;\;\text{in}\omega_{\varepsilon }. \end{array}$$

Proof: Starting with (10)–(12), we shall first prove the Boltzmann statistics (16) by introducing the entropy variables (the chemical potentials)

(17) $$\begin{array}{cc} & \mu_{s}^{\varepsilon }:=\ln c_{s}^{\varepsilon },\;s=0,\cdots ,n. \end{array}$$

Then, Equation (11) can be rewritten in terms of (17) in divergence form as

(18) $$\begin{array}{cc} & \int_{\Omega \backslash \partial \omega_{\varepsilon }}c_{s}^{\varepsilon }\nabla (\mu_{s}^{\varepsilon }+\chi_{{}_{\Omega \backslash \omega_{\varepsilon }}}\frac{z_{s}}{\kappa T}\phi^{\varepsilon }{)}^{⊤}D_{s}\nabla c_{s}\;dx=0,\;s=0,\cdots ,n,\\ & \;\;\text{for all test-functions}\;c\in H^{1}{\left(\Omega \backslash \partial \omega_{\varepsilon }\right)}^{n+1}:c=0\;\;\mathrm{on}\;\partial \Omega . \end{array}$$

Due to the boundary condition (10), we have $\phi^{\varepsilon }=0=\mu^{\varepsilon }$ on ∂Ω and the test-function $c_{s}=\mu_{s}^{\varepsilon }+\chi_{{}_{\Omega \backslash \omega_{\varepsilon }}}\frac{z_{s}}{\kappa T}\phi^{\varepsilon }$ can be inserted into (18). Hence, by recalling that $D_{s}$ are symmetric and positive definite matrices and $c^{\varepsilon }>0$, we derive the identity $\nabla (\mu_{s}^{\varepsilon }+\chi_{{}_{\Omega \backslash \omega_{\varepsilon }}}\frac{z_{s}}{\kappa T}\phi^{\varepsilon })=0,$s=0,⋯,n, a.e. in $\Omega \backslash \partial \omega_{\varepsilon }$. Using again the boundary condition (10), we conclude

(19) $$\begin{array}{c} \mu_{s}^{\varepsilon }+\chi_{{}_{\Omega \backslash \omega_{\varepsilon }}}\frac{z_{s}}{\kappa T}\phi^{\varepsilon }=0,\;s=0,\cdots ,n,\;\;\text{a.e. in}\Omega \backslash {\bar{\omega }}_{\varepsilon }, \end{array}$$

and $\mu_{s}^{\varepsilon }$ is an arbitrary constant in $\omega_{\varepsilon }$. This fact together with (17) implies (16). By substituting the expressions (16) into equation (12) and by using the charge-neutrality (2) on $\omega_{\varepsilon }$, equation (15b) follows directly.

Conversely, the equations (10)–(12) follow evidently from (15) and (16). This completes the proof. □

We remark that the concentrations $c^{\varepsilon }$ in (16) are unique up to fixing the constant positive values within the solid particles $\omega_{\varepsilon }$.

By exploiting Proposition 2, we construct a solution $\left(\phi^{\varepsilon },c^{\varepsilon }\right)$ for the variational problem (10)–(12) from the scalar problem (15) for the potential $\phi^{\varepsilon }$. The n+1 concentrations $c^{\varepsilon }$ are afterwards explicitly determined by (16).

Theorem 1: There exists the unique solution $\phi^{\varepsilon }$ to the semilinear problem (15) satisfying the following residual estimate

(20) $$\begin{array}{c} \|\nabla \phi^{\varepsilon }{\|}_{L^{2}\left(\Omega \backslash \partial \omega_{\varepsilon }\right)}^{2}+\frac{1}{\varepsilon }\|\left[\;\left[\phi^{\varepsilon }\right]\;\right]{\|}_{L^{2}\left(\partial \omega_{\varepsilon }\right)}^{2}+{\left\|\phi^{\varepsilon }\right\|}_{L^{2}\left(\Omega \backslash \omega_{\varepsilon }\right)}^{2}=O\left(1\right), \end{array}$$

which is uniform with respect to ε>0.

Proof: We first emphasise that for the first two terms on the left-hand side of (20) the following discontinuous version of Poincare’s inequality for homogeneous Dirichlet condition (15a) holds on the multiple domains $\Omega \backslash \partial \omega_{\varepsilon }$ without interfaces $\partial \omega_{\varepsilon }$ (see e.g. [34,36]):

(21) $$\begin{array}{c} K_{0}\|\phi^{\varepsilon }{\|}_{H^{1}\left(\Omega \backslash \partial \omega_{\varepsilon }\right)}^{2}\leq \|\nabla \phi^{\varepsilon }{\|}_{L^{2}\left(\Omega \backslash \partial \omega_{\varepsilon }\right)}^{2}+\frac{1}{\varepsilon }{\left\|\left[\;\left[\phi^{\varepsilon }\right]\;\right]\right\|}_{L^{2}\left(\partial \omega_{\varepsilon }\right)}^{2},\;\left(K_{0}>0\right). \end{array}$$

Therefore, the lower estimate (21) together with (3) ensures the coercivity of the operator of the problem (15b).

The main difficulty of the existence proof arises from the unbounded, exponential growth of the nonlinear term in (15b). While classic existence theorems on quasilinear equations are thus not applicable here, the solution can nevertheless be constructed by a thresholding, see e.g. [17] and references therein for the details.

To derive the estimate (20), it suffices to insert $\phi =\phi^{\varepsilon }$ as the test-function in the variational equation (15b) and apply (3) in order to estimate below the nonlinear term at the left-hand side of (15b). Finally the right-hand side of (15b) can be estimated by means of the following trace theorem

(22) $$\begin{array}{c} \int_{\partial \omega_{\varepsilon }^{-}}\varepsilon g\phi^{-}\;dS_{x}\leq {\left|g|\|\phi \right\|}_{H^{1}\left(\Omega \backslash \partial \omega_{\varepsilon }\right)}, \end{array}$$

see [30] for the details. This completes the proof. □

We remark that in the following Section 3, we will refine the residual error estimate (20) by means of asymptotic analysis as $\varepsilon ↘0^{+}$ and homogenisation.

## 3. Homogenisation and residual error estimate

We start the homogenisation procedure with three auxiliary cell problems. The first two cell problems serve to expand the inhomogeneous boundary traction g and the volume potential of the variational problem (15) from the porous space $\Omega \backslash {\bar{\omega }}_{\varepsilon }$ onto the whole domain $\Omega \backslash \partial \omega_{\varepsilon }$.

The third cell problem is needed to decompose the matrix $A^{\varepsilon }$ of oscillating coefficients in the cells with respect to small $\varepsilon ↘0^{+}$. This procedure will result in a regular asymptotic decomposition of the perturbation problem with a subsequent error estimate of the corrector term.

For a generic cell Υ, we introduce the Sobolev space $H_{\#}^{1}\left(\Upsilon \right)$ of functions which can be extended periodically to $H^{1}\left(R^{d}\right)$. This requires matching traces on the opposite faces of ∂Υ. Moreover, we shall denote by $H_{\#}^{1}\left(\Upsilon \backslash \partial \omega \right)$ those periodic functions, which are discontinuous, i.e. allow jumps across the interface ∂ω.

### 3.1. Auxiliary results

We state the first auxiliary cell problem as follows: find $L\in H^{1}\left(\Upsilon \backslash \partial \omega \right)$ such that

(23) $$\begin{array}{cc} \int_{\Upsilon \backslash \partial \omega }\left(\nabla L^{⊤}A\nabla u+Lu\right)\;dy= & \int_{\partial \omega^{-}}u^{-}\;dS_{y}\\ & \;\;\text{for all test-functions}\;u\in H^{1}\left(\Upsilon \backslash \partial \omega \right).\; \end{array}$$

In view of the homogenisation result stated in Theorem 2 in Section 3.2 below, the auxiliary problem (23) serves to expand the inhomogeneity of the boundary condition (8a) given by the material parameter g in terms of the weak formulation stated in (15b).

The existence of a unique solution L in (23) follows via standard elliptic theory from the assumed properties (6) of A. With its help, we are able to prove the following result.

Lemma 1: (The cell boundary-traction problem) For all test-functions $\phi \in H^{1}\left(\Omega \backslash \partial \omega_{\varepsilon }\right)$: ϕ=0 on ∂Ω holds the following expansion

(24) $$\begin{array}{c} \int_{\partial \omega_{\varepsilon }^{-}}\varepsilon g\phi^{-}\;dS_{x}-\int_{\Omega \backslash \partial \omega_{\varepsilon }}\frac{\left|\partial \omega \right|}{\left|\Upsilon \right|}g\phi \;dx=\varepsilon \;l_{1}\left(\phi \right), \end{array}$$

where $l_{1}:H^{1}\left(\Omega \backslash \partial \omega_{\varepsilon }\right)\mapsto R$ is a linear form satisfying

(25) $$\begin{array}{c} |l_{1}{\left(\phi \right)|\leq K\|\phi \|}_{H^{1}\left(\Omega \backslash \partial \omega_{\varepsilon }\right)},\;\left(K>0\right). \end{array}$$

Proof: We apply the auxiliary cell problem (23). By inserting a constant test-function u, we calculate the average value

(26) $$\begin{array}{c} {\left\langle L\right\rangle }_{y}=\frac{\left|\partial \omega \right|}{\left|\Upsilon \right|},\;\;\text{where}\;{\left\langle L\right\rangle }_{y}:=\frac{1}{\left|\Upsilon \right|}\int_{\Upsilon \backslash \partial \omega }L\;dy. \end{array}$$

Here, |∂ω| and |Υ| denote the Hausdorff measures of the solid particle boundary ∂ω in $R^{d-1}$ and of the cell Υ in $R^{d}$, respectively.

Subtracting $\int_{\Upsilon \backslash \partial \omega }{\left\langle L\right\rangle }_{y}u\;dy$ from (23), we rewrite it equivalently as

(27) $$\begin{array}{cc} & \int_{\partial \omega^{-}}u^{-}\;dS_{y}-\int_{\Upsilon \backslash \partial \omega }{\left\langle L\right\rangle }_{y}u\;dy\\ & \;=\int_{\Upsilon \backslash \partial \omega }(\nabla_{\;y}L^{⊤}A\nabla_{\;y}u+\left(L-{\left\langle L\right\rangle }_{y}\right)\left(u-{\left\langle u\right\rangle }_{y}\right))\;dy=:l\left(u\right), \end{array}$$

where we have added to the residuum l(u) the trivial term

$$\int_{\Upsilon \backslash \partial \omega }\left(L-{\left\langle L\right\rangle }_{y}\right){\left\langle u\right\rangle }_{y}\;dy=0,\;{\left\langle u\right\rangle }_{y}:=\frac{1}{\left|\Upsilon \right|}\int_{\Upsilon \backslash \partial \omega }u\;dy.$$

In the following, we shall apply the discontinuous Poincare inequality

(28) $$\begin{array}{c} K_{1}{\|u-\left\langle u\right\rangle }_{y}{\|}_{L^{2}\left(\Upsilon \backslash \partial \omega \right)}\leq \|\nabla_{\;y}{u\|}_{L^{2}\left(\Upsilon \backslash \partial \omega \right)}+{\left\|\left[\;\left[u\right]\;\right]\right\|}_{L^{2}\left(\partial \omega \right)},\;\left(K_{1}>0\right), \end{array}$$

and the Trace Theorem

(29) $$\begin{array}{c} {\left\|\left[\;\left[u\right]\;\right]\right\|}_{L^{2}\left(\partial \omega \right)}\leq \frac{K_{2}}{\sqrt{2}}(\|\nabla_{\;y}{u\|}_{L^{2}\left(\Upsilon \backslash \partial \omega \right)}+{\left\|u\right\|}_{L^{2}\left(\Upsilon \backslash \partial \omega \right)})\leq K_{2}{\left\|u\right\|}_{H^{1}\left(\Upsilon \backslash \partial \omega \right)}, \end{array}$$

with $K_{2}>0$, which combine to the estimate

(30) $$\begin{array}{c} {\|u-\left\langle u\right\rangle }_{y}{\|}_{L^{2}\left(\Upsilon \backslash \partial \omega \right)}\leq K_{3}{\left\|u\right\|}_{H^{1}\left(\Upsilon \backslash \partial \omega \right)},\;\left(K_{3}=K_{1}^{-1}\left(1+K_{2}\right)\right). \end{array}$$

By recalling that $A\in L^{\infty }{\left(\Upsilon \right)}^{d\times d}$ and by applying Cauchy’s inequality to the right-hand side of (27) and subsequently applying estimate (30) to L and u, we obtain the following estimate

(31) $$\begin{array}{cc} \left|l(u)\right| & \leq \bar{K}{\left\|\nabla L\right\|}_{L^{2}\left(\Upsilon \backslash \partial \omega \right)}{\left\|\nabla u\right\|}_{L^{2}\left(\Upsilon \backslash \partial \omega \right)}+K_{3}^{2}{\left\|L\right\|}_{H^{1}\left(\Upsilon \backslash \partial \omega \right)}{\left\|u\right\|}_{H^{1}\left(\Upsilon \backslash \partial \omega \right)}\\ & \leq \left(\bar{K}+K_{3}^{2}\right){\left\|L\right\|}_{H^{1}\left(\Upsilon \backslash \partial \omega \right)}{\left\|u\right\|}_{H^{1}\left(\Upsilon \backslash \partial \omega \right)}, \end{array}$$

with $\bar{K}$ from (6) and $K_{3}$ from (30).

For a proper test-function ϕ(x) with $x=\varepsilon \lfloor \frac{x}{\varepsilon }\rfloor +\varepsilon \left\{\frac{x}{\varepsilon }\right\}$, we insert $u\left(x,y\right)=\phi \left(\varepsilon \lfloor \frac{x}{\varepsilon }\rfloor +\varepsilon y\right)$ into (27) and apply the periodic coordinate transformation y↦x, $\Upsilon \mapsto R^{d}$, by paving $R^{d}$ such that $\{\frac{x}{\varepsilon }\}=y$ (recall Section 2.1). After observing that $dy\mapsto \varepsilon^{-d}dx$, $dS_{y}\mapsto \varepsilon^{1-d}dS_{x}$, $\nabla_{y}\mapsto \varepsilon \nabla_{x}$, we also multiply (27) with the constant $g\varepsilon^{d}$ and use (26) in order to derive

$$\begin{array}{c} \sum_{p=1}^{N_{\varepsilon }}\int_{{\left(\partial \omega_{p}^{\varepsilon }\right)}^{-}}\varepsilon g\phi^{-}\;dS_{x}-\sum_{p=1}^{N_{\varepsilon }}\int_{\Upsilon_{p}^{\varepsilon }\backslash \partial \omega_{p}^{\varepsilon }}\frac{\left|\partial \omega \right|}{\left|\Upsilon \right|}g\phi \;dx=\varepsilon \;l_{1}\left(\phi \right), \end{array}$$

which is (24) with the following right-hand side term:

(32) $$\begin{array}{c} l_{1}\left(\phi \right):=g\sum_{p=1}^{N_{\varepsilon }}\int_{\Upsilon_{p}^{\varepsilon }\backslash \partial \omega_{p}^{\varepsilon }}({\left(\varepsilon \nabla_{\;x}L^{\varepsilon }\right)}^{⊤}A^{\varepsilon }\nabla_{\;x}\phi +\left(L^{\varepsilon }-{\left\langle L\right\rangle }_{y}\right)\cdot \frac{1}{\varepsilon }\left(\phi -{\left\langle \phi \right\rangle }_{y}\right))\;dx, \end{array}$$

where we denote $L^{\varepsilon }\left(x\right):=L\left(\left\{\frac{x}{\varepsilon }\right\}\right)$ and $A^{\varepsilon }\left(x\right):=A\left(\left\{\frac{x}{\varepsilon }\right\}\right)$.

Similarly, (30) transforms into the uniform estimate

(33) $$\begin{array}{cc} \frac{1}{\varepsilon }{\left\|\phi -{\left\langle \phi \right\rangle }_{y}\right\|}_{L^{2}\left(\Upsilon_{p}^{\varepsilon }\backslash \partial \omega_{p}^{\varepsilon }\right)} & \leq K_{3}(\|\nabla_{\;x}{\phi \|}_{L^{2}\left(\Upsilon_{p}^{\varepsilon }\backslash \partial \omega_{p}^{\varepsilon }\right)}+\frac{1}{\varepsilon }{\left\|\phi \right\|}_{L^{2}\left(\Upsilon_{p}^{\varepsilon }\backslash \partial \omega_{p}^{\varepsilon }\right)})\\ & \leq K_{3}{\left\|\phi \right\|}_{H^{1}\left(\Upsilon_{p}^{\varepsilon }\backslash \partial \omega_{p}^{\varepsilon }\right)}, \end{array}$$

with $K_{3}>0$ from (30). We note that the first line of (33) expresses the $H^{1}$-norm by the standard homogeneity argument, see e.g. [22, Appendix, Lemma 1, p.370].

Therefore, the estimate (31) of l yields the following estimate of $l_{1}$

(34) $$\begin{array}{cc} |l_{1}\left(\phi \right)| & \leq \left|g\right|\sum_{p=1}^{N_{\varepsilon }}(\bar{K}\|\nabla_{y}{L\|}_{L^{2}\left(\Upsilon \backslash \partial \omega \right)}{\left\|\nabla_{x}\phi \right\|}_{L^{2}\left(\Upsilon_{p}^{\varepsilon }\backslash \partial \omega_{p}^{\varepsilon }\right)}\\ & {\;+\|L-\left\langle L\right\rangle }_{y}{\|}_{L^{2}\left(\Upsilon \backslash \partial \omega \right)}\cdot \frac{1}{\varepsilon }{\left\|\phi -{\left\langle \phi \right\rangle }_{y}\right\|}_{L^{2}\left(\Upsilon_{p}^{\varepsilon }\backslash \partial \omega_{p}^{\varepsilon }\right)})\\ & \leq \left|g\right|\left(\bar{K}+K_{3}^{2}\right){\left\|L\right\|}_{H^{1}\left(\Upsilon \backslash \partial \omega \right)}\sum_{p=1}^{N_{\varepsilon }}{\left\|\phi \right\|}_{H^{1}\left(\Upsilon_{p}^{\varepsilon }\backslash \partial \omega_{p}^{\varepsilon }\right)}. \end{array}$$

Here we used (6) and inequalities (30) for L and (33) for ϕ. Then, (34) follows (25) with the constant $K=\left|g\right|\left(\bar{K}+K_{3}^{2}\right){\left\|L\right\|}_{H^{1}\left(\Upsilon \backslash \partial \omega \right)}$, which completes the proof. □

Remark 1: We remark that Lemma 1 justifies not only the a-priori estimate (22), but also refines it by specifying the limiting asymptotic term as $\varepsilon ↘0^{+}$, which consists of the constant potential $\frac{\left|\partial \omega \right|}{\left|\Upsilon \right|}g$ distributed uniformly over Ω.

Another idea for the proof of Lemma 1 can be referred in [30,33].

The next auxiliary cell problem studies the asymptotic expansion of a volume force $f\in H^{1}\left(\Omega \backslash \partial \omega_{\varepsilon }\right)$, which is given on the porous space $\Upsilon \backslash \bar{\omega }$ surrounding the solid particle ω⊂Υ. It will be applied in particular to the nonlinear term in (15b), i.e. we shall consider the specific volume force $f\left(x\right)=-\sum_{s=0}^{n}z_{s}\exp\left(-\frac{z_{s}}{\kappa T}\phi^{0}\left(x\right)\right)$ in Theorem 2 below.

With $x=\varepsilon \left\lfloor \frac{x}{\varepsilon }\right\rfloor +\varepsilon \left\{\frac{x}{\varepsilon }\right\}$ (recall Section 2.1), the following unfolding operator

$$T_{\varepsilon }:\left\{\begin{array}{c} H^{1}\left(\Omega \backslash \partial \omega_{\varepsilon }\right)\mapsto L^{2}(\Omega ,H^{1}\left(\Upsilon \backslash \partial \omega \right)),\\ \left[1mm\right]\left(T_{\varepsilon }f\right)\left(x,y\right):=f\left(\varepsilon \lfloor \frac{x}{\varepsilon }\rfloor +\varepsilon y\right), \end{array}\right.$$

is well defined, see [26]. For its modification near the boundaries ∂Ω of nonrectangular domains Ω, see [40].

For $x\in \Omega \backslash \partial \omega_{\varepsilon }$, there exists a function M(x,y) piecewisely composed of solutions M(x,·) of the following x-dependent cell problems (compare with (23)): find $M\left(x,\;\cdot \;\right)\in H^{1}\left(\Upsilon \backslash \partial \omega \right)$ such that

(35) $$\begin{array}{cc} \int_{\Upsilon \backslash \partial \omega }\left(\nabla_{\;y}M^{⊤}A\nabla_{\;y}u+Mu\right)\;dy= & \int_{\Upsilon \backslash \omega }\left(T_{\varepsilon }f\right)\;u\;dy\\ & \;\;\text{for all test-functions}\;u\in H^{1}\left(\Upsilon \backslash \partial \omega \right). \end{array}$$

Lemma 2: (Unfolding of the cell volume-force problem) For all $\phi \in H^{1}\left(\Omega \backslash \partial \omega_{\varepsilon }\right)$: ϕ=0 on ∂Ω holds the following expansion

(36) $$\begin{array}{c} \int_{\Omega \backslash \omega_{\varepsilon }}f\phi \;dx-\frac{\left|\Upsilon \backslash \omega \right|}{\left|\Upsilon \right|}\int_{\Omega \backslash \partial \omega_{\varepsilon }}f\phi \;dx=\varepsilon \;l_{2}\left(\phi \right), \end{array}$$

where $l_{2}:H^{1}\left(\Omega \backslash \partial \omega_{\varepsilon }\right)\mapsto R$ is a linear form satisfying

(37) $$\begin{array}{c} |l_{2}{\left(\phi \right)|\leq K\|\phi \|}_{H^{1}\left(\Omega \backslash \partial \omega_{\varepsilon }\right)},\;\left(K>0\right). \end{array}$$

Proof: By inserting a constant test-function u into the auxiliary cell problem (35), we obtain the locally averaged value of M=M(x,y)

(38) $$\begin{array}{c} \langle M\left(x,\;\cdot \;\right)\rangle_{y}:=\frac{1}{\left|\Upsilon \right|}\int_{\Upsilon \backslash \partial \omega }M\;dy=\frac{1}{\left|\Upsilon \right|}\int_{\Upsilon \backslash \omega }T_{\varepsilon }f\;dy. \end{array}$$

Moreover, by using the average $\langle T_{\varepsilon }f\rangle_{y}$, we can expand

(39) $$\begin{array}{c} F\left(x,y\right):=\left(T_{\varepsilon }f\right)\left(x,y\right)-\langle T_{\varepsilon }f\rangle_{y},\;\langle T_{\varepsilon }f\rangle_{y}:=\frac{1}{\left|\Upsilon \right|}\int_{\Upsilon \backslash \partial \omega }\left(T_{\varepsilon }f\right)\left(x,\cdot \right)\;dy. \end{array}$$

See [41] for the analysis of expansion (39) in terms of Fourier series. For fixed x the residual F(x,y) has zero average ${\left\langle F\right\rangle }_{y}=0$ and estimates as

(40) $$\begin{array}{c} {\left\|F\left(x,\;\cdot \;\right)\right\|}_{L^{2}\left(\Upsilon \backslash \partial \omega \right)}=\|T_{\varepsilon }f-{\left\langle T_{\varepsilon }f\right\rangle }_{y}{\|}_{L^{2}\left(\Upsilon \backslash \partial \omega \right)}\leq K_{3}{\left\|T_{\varepsilon }f\right\|}_{H^{1}\left(\Upsilon \backslash \partial \omega \right)} \end{array}$$

due to the discontinuous Poincare inequality (30). By inserting (39) into (38), we calculate

$$\begin{array}{c} {\left\langle M\right\rangle }_{y}=\frac{1}{\left|\Upsilon \right|}\int_{\Upsilon \backslash \omega }T_{\varepsilon }f\;dy=\frac{1}{\left|\Upsilon \right|}\int_{\Upsilon \backslash \omega }F\;dy+\frac{\left|\Upsilon \backslash \omega \right|}{\left|\Upsilon \right|}\;{\left\langle T_{\varepsilon }f\right\rangle }_{y}, \end{array}$$

and thus derive by using again (39), i.e. ${\left\langle T_{\varepsilon }f\right\rangle }_{y}=T_{\varepsilon }f-F$

(41) $$\begin{array}{c} \frac{\left|\Upsilon \backslash \omega \right|}{\left|\Upsilon \right|}\;T_{\varepsilon }f={\left\langle M\right\rangle }_{y}+\frac{\left|\Upsilon \backslash \omega \right|}{\left|\Upsilon \right|}(F-\frac{1}{\left|\Upsilon \backslash \omega \right|}\int_{\Upsilon \backslash \omega }F\;dy). \end{array}$$

After multiplying the identity (41) with u and integrating it over Υ\∂ω, we subtract it from (35) and rewrite (35) equivalently as

(42) $$\begin{array}{cc} & \int_{\Upsilon \backslash \omega }\left(T_{\varepsilon }f\right)\;u\;dy-\frac{\left|\Upsilon \backslash \omega \right|}{\left|\Upsilon \right|}\int_{\Upsilon \backslash \partial \omega }\left(T_{\varepsilon }f\right)\;u\;dy\\ & \;=-\frac{\left|\Upsilon \backslash \omega \right|}{\left|\Upsilon \right|}\int_{\Upsilon \backslash \partial \omega }(F-\frac{1}{\left|\Upsilon \backslash \omega \right|}\int_{\Upsilon \backslash \omega }F\;dy)u\;dy\\ & \;+\int_{\Upsilon \backslash \partial \omega }(\nabla_{\;y}M^{⊤}A\nabla_{\;y}u+\left(M-{\left\langle M\right\rangle }_{y}\right)\left(u-{\left\langle u\right\rangle }_{y}\right))\;dy=:m\left(u\right), \end{array}$$

where we have added the trivial term $\int_{\Upsilon \backslash \partial \omega }\left(M-{\left\langle M\right\rangle }_{y}\right){\left\langle u\right\rangle }_{y}\;dy=0$ and the residuum m(u) shortly denotes the right-hand side terms of (42).

For fixed $x\in \Omega \backslash \partial \omega_{\varepsilon }$, Cauchy’s inequality yields for the first term on the right-hand side of (42)

(43) $$\begin{array}{cc} & |\int_{\Upsilon \backslash \partial \omega }(F-\frac{1}{\left|\Upsilon \backslash \omega \right|}\int_{\Upsilon \backslash \omega }F\;dy)u\;dy|\\ & \;\leq {\left\|F\right\|}_{L^{2}\left(\Upsilon \backslash \partial \omega \right)}{\left\|u\right\|}_{L^{2}\left(\Upsilon \backslash \partial \omega \right)}+\sqrt{\frac{\left|\Upsilon \right|}{\left|\Upsilon \backslash \omega \right|}}{\;\|F\|}_{L^{2}\left(\Upsilon \backslash \omega \right)}\;{\left\|u\right\|}_{L^{2}\left(\Upsilon \backslash \partial \omega \right)}\\ & \;\leq (1+\sqrt{\frac{\left|\Upsilon \right|}{\left|\Upsilon \backslash \omega \right|}}{)\|F\|}_{L^{2}\left(\Upsilon \backslash \partial \omega \right)}{\left\|u\right\|}_{L^{2}\left(\Upsilon \backslash \partial \omega \right)}. \end{array}$$

Thus, by applying the estimates (40) and (43) to F and the discontinuous Poincare inequality (30) to M and u, we estimate m(u) at the right-hand side of (42) as

(44) $$\begin{array}{cc} & \left|m\left(u\right)\right|\leq K_{4}\|T_{\varepsilon }{f\|}_{H^{1}\left(\Upsilon \backslash \partial \omega \right)}{\left\|u\right\|}_{L^{2}\left(\Upsilon \backslash \partial \omega \right)}\\ & \;+\left(\bar{K}+K_{3}^{2}\right){\left\|M\right\|}_{H^{1}\left(\Upsilon \backslash \partial \omega \right)}{\left\|u\right\|}_{H^{1}\left(\Upsilon \backslash \partial \omega \right)}, \end{array}$$

where $K_{4}=\frac{\left|\Upsilon \backslash \omega \right|}{\left|\Upsilon \right|}(1+\sqrt{\frac{\left|\Upsilon \right|}{\left|\Upsilon \backslash \omega \right|}})K_{3}$ and by recalling $\bar{K}$ from (6) and $K_{3}$ from (30).

Next, we substitute $u=(T_{\varepsilon }\phi )$ as the test-function in (42) and use the property $T_{\varepsilon }f\cdot T_{\varepsilon }\phi =T_{\varepsilon }\left(f\phi \right)$ of the unfolding operator. After applying the periodic coordinate transformation y↦x, $\{\frac{x}{\varepsilon }\}=y$ to (42) similar to the proof of Lemma 1, we arrive with $T_{\varepsilon }\left(f\phi \right)\mapsto f\phi$ and $T_{\varepsilon }\phi \mapsto \phi$ at (36) with

(45) $$\begin{array}{cc} l_{2}\left(\phi \right):= & \sum_{p=1}^{N_{\varepsilon }}[\frac{1}{\varepsilon }\frac{\left|\Upsilon \backslash \omega \right|}{\left|\Upsilon \right|}\int_{\Upsilon_{p}^{\varepsilon }\backslash \partial \omega_{p}^{\varepsilon }}(F^{\varepsilon }-\frac{1}{\left|\Upsilon \backslash \omega \right|}\int_{\Upsilon \backslash \omega }F\;dy)\phi \;dx\\ & \;+\int_{\Upsilon_{p}^{\varepsilon }\backslash \partial \omega_{p}^{\varepsilon }}({\left(\varepsilon \nabla_{\;x}M^{\varepsilon }\right)}^{⊤}A^{\varepsilon }\nabla_{\;x}\phi +\left(M^{\varepsilon }-{\left\langle M\right\rangle }_{y}\right)\frac{1}{\varepsilon }\left(\phi -{\left\langle T_{\varepsilon }\phi \right\rangle }_{y}\right))\;dx], \end{array}$$

where $F^{\varepsilon }\left(x\right):=F\left(x,\left\{\frac{x}{\varepsilon }\right\}\right)$ and $M^{\varepsilon }\left(x\right):=M\left(x,\left\{\frac{x}{\varepsilon }\right\}\right)$. Similarly to (44), we estimate with $F^{\varepsilon }\left(x\right)=f\left(x\right)-{\left\langle T_{\varepsilon }f\right\rangle }_{y}\left(x\right)$

$$\begin{array}{cc} |l_{2}\left(\phi \right)|\leq & \;\sum_{p=1}^{N_{\varepsilon }}[\frac{\left|\Upsilon \backslash \omega \right|}{\left|\Upsilon \right|}(1\;+\;\sqrt{\frac{\left|\Upsilon \right|}{\left|\Upsilon \backslash \omega \right|}})\frac{1}{\varepsilon }\|f-{\left\langle T_{\varepsilon }f\right\rangle }_{y}{\|}_{L^{2}\left(\Upsilon_{p}^{\varepsilon }\backslash \partial \omega_{p}^{\varepsilon }\right)}{\left\|\phi \right\|}_{L^{2}\left(\Upsilon_{p}^{\varepsilon }\backslash \partial \omega_{p}^{\varepsilon }\right)}\\ & +\underset{x\in \Omega \backslash \partial \omega_{\varepsilon }}{\sup}\;\;\{\bar{K}\|\nabla_{\;y}{M\left(x,\;\cdot \;\right)\|}_{L^{2}\left(\Upsilon \backslash \partial \omega \right)}{\left\|\nabla \phi \right\|}_{L^{2}\left(\Upsilon_{p}^{\varepsilon }\backslash \partial \omega_{p}^{\varepsilon }\right)}\\ & {+\|M\left(x,\;\cdot \;\right)-\left\langle M\left(x,\;\cdot \;\right)\right\rangle }_{y}{\|}_{L^{2}\left(\Upsilon \backslash \partial \omega \right)}\frac{1}{\varepsilon }{\left\|\phi -{\left\langle T_{\varepsilon }\phi \right\rangle }_{y}\right\|}_{L^{2}\left(\Upsilon_{p}^{\varepsilon }\backslash \partial \omega_{p}^{\varepsilon }\right)}\}], \end{array}$$

hence,

(46) $$\begin{array}{cc} |l_{2}\left(\phi \right)|\leq & \;K_{4}{\left\|f\right\|}_{H^{1}\left(\Omega \backslash \partial \omega_{\varepsilon }\right)}{\left\|\phi \right\|}_{L^{2}\left(\Omega \backslash \partial \omega_{\varepsilon }\right)}\\ & \;+\left(\bar{K}+K_{3}^{2}\right)\underset{x\in \Omega \backslash \partial \omega_{\varepsilon }}{\sup}\|M\left(x,\;\cdot \;\right){\|}_{H^{1}\left(\Upsilon \backslash \partial \omega \right)}{\left\|\phi \right\|}_{H^{1}\left(\Omega \backslash \partial \omega_{\varepsilon }\right)}, \end{array}$$

where we have used (30) for M(x,·) and (33) for f and ϕ. Thus, (46) implies the estimate (37) of the residual term $l_{2}$ given in (45) with

$$K=K_{4}{\left\|f\right\|}_{H^{1}\left(\Omega \backslash \partial \omega_{\varepsilon }\right)}+\left(\bar{K}+K_{3}^{2}\right)\;\;\;\underset{x\in \Omega \backslash \partial \omega_{\varepsilon }}{\sup}\;\;\;{\left\|M\left(x,\;\cdot \;\right)\right\|}_{H^{1}\left(\Upsilon \backslash \partial \omega \right)}.$$

This completes the proof. □

Remark 2: We remark that the factor $\frac{\left|\Upsilon \backslash \omega \right|}{\left|\Upsilon \right|}$ in (36) reflects the porosity of the cell Υ due to the presence of the solid particles ω. In our particular geometric setting, we have |Υ|=1 and |Υ\ω|=1-|ω|, respectively.

The third cell problem considers the solutions of the following system of d linear equations: find a vector of periodic functions $N={\left(N_{1},\cdots ,N_{d}\right)}^{⊤}\in H_{\#}^{1}{\left(\Upsilon \backslash \partial \omega \right)}^{d}$ with componentwise zero average ${\left\langle N\right\rangle }_{y}=0$ such that

(47) $$\begin{array}{cc} & \int_{\Upsilon \backslash \partial \omega }D\left(N+y\right)A\nabla u\;dy+\int_{\partial \omega }\alpha \left[\;\left[N\right]\;\right]\left[\;\left[u\right]\;\right]\;dS_{y}=0,\\ & \;\;\text{for all scalar test-functions}u\in H_{\#}^{1}\left(\Upsilon \backslash \partial \omega \right). \end{array}$$

Here, $H_{\#}^{1}\left(\Upsilon \backslash \partial \omega \right)$ denotes the space of periodic $H^{1}$-functions and $DN\left(y\right)\in R^{d\times d}$ for y∈Υ\∂ω stands for the row-wise gradient matrix of the vector N, that is

$$DN:=\left(\begin{array}{ccc} N_{1,1} & \cdots & N_{1,d}\\ \vdots & \; & \vdots \\ N_{d,1} & \cdots & N_{d,d} \end{array}\right),\;\;\text{where}\;N_{i,j}:=\frac{\partial N_{i}}{\partial y_{j}},\;i,j=1,\cdots ,d.$$

Moreover in (47), $Dy=I\in R^{d\times d}$ yields the identity matrix. The solvability of (47) follows from the symmetry and positive definiteness assumption (6). The uniqueness of the solution N is provided due to the constraint ${\left\langle N\right\rangle }_{y}=0$. Indeed, since N(y)+K with an arbitrary constant K solves also (47), the zero average condition is sufficient (and necessary) to ensure the uniqueness of the solution, see e.g. [21]. Finally, the solution is smooth locally in Υ\∂ω.

Remark 3: We remark in particular, that if [[N]]=[[u]]=0 would hold, then the discontinuous cell problem (47) would reduce to a standard, continuous cell problem.

The system (47) is essential to determine the efficient coefficient matrix $A^{0}$ of the macroscopic model averaged over Ω. In fact, following the lines of [21,23], we shall establish an orthogonal decomposition of Helmholtz type for the oscillating coefficients $A^{\varepsilon }$.

The Helmholtz type decomposition is based on the left-hand side of (47) defining an inner product ⟨⟨·,·⟩⟩ in $H_{\#}^{1}\left(\Upsilon \backslash \partial \omega \right)$. Due to [[y]]=0, the variational equation (47) reads as ⟨⟨N+y,u⟩⟩=0 for all $u\in H_{\#}^{1}\left(\Upsilon \backslash \partial \omega \right)$, which implies that N+y belongs to the kernel of this topological vector space. Thus, the fundamental theorem of vector calculus (the Helmholtz theorem, see e.g. [23]) permits the following representation as sum of a constant matrix $A^{0}$ and divergence free B(y) fields in $R^{d\times d}$:

(48) $$\begin{array}{c} D(N\left(y\right)+y)A\left(y\right)=A^{0}+B\left(y\right),\;\;\text{a.e.}\;y\in \Upsilon \backslash \partial \omega , \end{array}$$

where B has zero average, i.e.

$$0={\left\langle B\right\rangle }_{y}:=\frac{1}{\left|\Upsilon \right|}\int_{\Upsilon \backslash \partial \omega }B\left(y\right)\;dy.$$

Thus, we obtain the following lemma:

Lemma 3: (The cell oscillating-coefficient problem) The constant matrix of effective coefficients is determined by averaging

(49) $$\begin{array}{c} A^{0}:=\langle D(N\left(y\right)+y)A\rangle_{y}\in R^{d\times d}. \end{array}$$

Moreover, $A^{0}$ is a symmetric and positive definite matrix with the entries:

(50) $$\begin{array}{cc} A_{ij}^{0}= & \langle \sum_{k,l=1}^{d}\left(N_{i,k}+\delta_{i,k}\right)A_{kl}\left(N_{j,l}+\delta_{j,l}\right)\rangle_{\;y}+\frac{1}{\left|\Upsilon \right|}\int_{\partial \omega }\alpha \left[\;\left[N_{i}\right]\;\right]\left[\;\left[N_{j}\right]\;\right]\;dS_{y}\\ & \;\;\text{for}\;i,j=1,\cdots ,d. \end{array}$$

For the transformed solution vector $N^{\varepsilon }\left(x\right):=N\left(\left\{\frac{x}{\varepsilon }\right\}\right)$, which depends only on $\left\{\frac{x}{\varepsilon }\right\}$ since the coefficient $A^{\varepsilon }\left(x\right):=A\left(\left\{\frac{x}{\varepsilon }\right\}\right)$ also depends only on $\left\{\frac{x}{\varepsilon }\right\}$, the following decomposition holds:

(51) $$\begin{array}{c} D\left(\varepsilon N^{\varepsilon }\left(x\right)+x\right)A^{\varepsilon }\left(x\right)=A^{0}+\varepsilon B^{\varepsilon }\left(x\right)\;\;\text{in}R^{d\times d}\;\;\text{and}\;\;\text{a.e.}\;x\in \Omega \backslash \partial \omega_{\varepsilon }. \end{array}$$

The transformed function $B^{\varepsilon }\left(x\right):=B\left(\left\{\frac{x}{\varepsilon }\right\}\right)$ is deduced from the symmetric matrix $B\in L_{\mathrm{div}}^{2}{\left(\Upsilon \backslash \partial \omega \right)}^{d\times d}$ with zero average ${\left\langle B\right\rangle }_{y}=0$. Its entries $B_{ij}\left(y\right)$, i,j=1,⋯,d express divergence free fields (called solenoidal in 3d) obtained by combining the derivatives $\frac{\partial }{\partial y_{k}}$, k=1,⋯,d of a third-order skew-symmetric tensor $b_{ijk}$ in the following way

(52) $$\begin{array}{c} B_{ij}=\sum_{k=1}^{d}b_{ijk,k},\;b_{ijk}=-b_{ikj},\;\text{(skew-symmetry)}\;\;\text{a.e. on}\Upsilon \backslash \partial \omega . \end{array}$$

It follows in particular from (52) that

(53) $$\begin{array}{c} \sum_{j,k=1}^{d}b_{ijk}=0,\;\sum_{j=1}^{d}B_{ij,j}=0,\;i=1,\cdots ,d\;\;\text{a.e. on}\Upsilon \backslash \partial \omega . \end{array}$$

At the interface the following jump relations hold:

(54) $$\begin{array}{c} \left[\left[B^{\varepsilon }\right]\right]=0,\;\left(A^{0}+\varepsilon B^{\varepsilon }\right)\nu =\alpha \left[\left[N^{\varepsilon }\right]\right]\;\;\text{a.e. on}\partial \omega_{\varepsilon }. \end{array}$$

Proof: The constant values of $A^{0}$ stated in (49) follow from averaging (48) with ${\left\langle \;\cdot \;\right\rangle }_{y}$ over Υ\∂ω and by using ${\left\langle B\right\rangle }_{y}=0$. The formula (50) can be checked directly from the ith component of equation (47) tested with the function $u=N_{j}$, divided by |Υ|=|Υ\∂ω|, and from (49) for i,j=1,⋯,d:

$$\begin{array}{cc} & \langle \sum_{k,l=1}^{d}\left(N_{i,k}+\delta_{i,k}\right)A_{kl}\left(N_{j,l}+\delta_{j,l}\right)\rangle_{\;y}=\langle \sum_{k,l=1}^{d}\left(N_{i,k}+\delta_{i,k}\right)A_{kl}N_{j,l}\rangle_{\;y}\\ & \;+\langle \sum_{k=1}^{d}\left(N_{i,k}+\delta_{i,k}\right)A_{kj}\rangle_{\;y}=-\frac{1}{\left|\Upsilon \right|}\int_{\partial \omega }\alpha \left[\;\left[N_{i}\right]\;\right]\left[\;\left[N_{j}\right]\;\right]\;dS_{y}+A_{ij}^{0}. \end{array}$$

The symmetry and positive definiteness of $A^{0}$ follow straightforward from the assumption in (6) of A being symmetric and positive definite. The formulas (52) and (53) describe the fact that the columns of B are divergence free. Inserting the representation (48) into (47) and integrating by parts yields

$$\begin{array}{cc} 0 & =\int_{\Upsilon \backslash \partial \omega }\left(A^{0}+B\right)\nabla u\;dy+\int_{\partial \omega }\alpha \left[\;\left[N\right]\;\right]\left[\;\left[u\right]\;\right]\;dS_{y}\\ & =\int_{\partial \omega }(\alpha \left[\;\left[N\right]\;\right]\left[\;\left[u\right]\;\right]-\left[\;\left[\left(A^{0}+B\right)\nu \;u\right]\;\right])\;dS_{y} \end{array}$$

due to the second equality in (53). Then, by choosing test-functions $u\in H_{\#}^{1}\left(\Upsilon \backslash \partial \omega \right)$ satisfying either [[u]]=0 or [[u]]≠0, it follows

(55) $$\begin{array}{c} \left[\left[B\right]\right]=0,\;\left(A^{0}+B\right)\nu =\alpha \left[\left[N\right]\right]\;\;\text{a.e.}\;\;\text{on}\;\partial \omega . \end{array}$$

Finally, we apply the periodic coordinate transformation y↦x, $\Upsilon \mapsto R^{d}$, with $y=\{\frac{x}{\varepsilon }\}$ to (48) and (55). With $\nabla_{\;y}\mapsto \varepsilon \nabla_{\;x}$, we have for the row-wise gradient matrix $D_{y}N\mapsto \varepsilon D_{x}N^{\varepsilon }$ and $B\mapsto \varepsilon B^{\varepsilon }$. Thus, we arrive at (51) and (54). The proof is completed. □

### 3.2. The main theorem

Based on the Lemmata 1–3, we formulate the main homogenisation result:

Theorem 2: The homogenisation of the discontinuous nonlinear PB problem under the interfacial jump conditions (15) yields the following averaged (macroscopic) nonlinear PB problem: find $\phi^{0}\in H_{0}^{1}\left(\Omega \right)$ such that

(56) $$\begin{array}{cc} \int_{\Omega }({\left(\nabla \phi^{0}\right)}^{⊤}A^{0}\nabla \phi -\frac{\left|\Upsilon \backslash \omega \right|}{\left|\Upsilon \right|}\sum_{s=0}^{n}z_{s}e^{-\frac{z_{s}}{\kappa T}\phi^{0}}\phi )\;dx= & \int_{\Omega }\frac{\left|\partial \omega \right|}{\left|\Upsilon \right|}g\phi \;dx\\ & \;\;\text{for all test-functions}\;\phi \in H_{0}^{1}\left(\Omega \right). \end{array}$$

For smooth solutions $\phi^{0}$ and N of (56) and (47), in the limit $\varepsilon ↘0^{+}$, the solution $\phi^{\varepsilon }$ of (15) and the corrector term $\phi^{1}:=\phi^{0}+\varepsilon {\left(\nabla \phi^{0}\right)}^{⊤}N^{\varepsilon }$ to $\phi^{0}$ satisfy the residual error estimate (improving (20)):

(57) $$\begin{array}{c} \|\nabla \left(\phi^{\varepsilon }-\phi^{1}\right){\|}_{L^{2}\left(\Omega \backslash \partial \omega_{\varepsilon }\right)}^{2}+\frac{1}{\varepsilon }{\left\|\left[\;\left[\phi^{\varepsilon }-\phi^{1}\right]\;\right]\right\|}_{L^{2}\left(\partial \omega_{\varepsilon }\right)}^{2}=O\left(\varepsilon \right). \end{array}$$

Proof: First, we remark that the left-hand side of (57) defines a norm in $H^{1}\left(\Omega \backslash \partial \omega_{\varepsilon }\right)$ due to the lower estimate (21).

Secondly, the unique solution $\phi^{0}$ of (56) can be established by following the arguments given in the proof of Theorem 1. Moreover, the solution is smooth inside Ω by standard arguments of local regularity of weak solutions, see e.g. [22, Appendix] and references therein.

Next, we prove the residual error estimate (57). Integrating (56) by parts on Ω yields the strong formulation

(58) $$\begin{array}{c} -\mathrm{div}(A^{0}\nabla \phi^{0})-\frac{\left|\Upsilon \backslash \omega \right|}{\left|\Upsilon \right|}\sum_{s=0}^{n}z_{s}e^{-\frac{z_{s}}{\kappa T}\phi^{0}}=\frac{\left|\partial \omega \right|}{\left|\Upsilon \right|}g,\;\;\text{in}\Omega . \end{array}$$

By applying the Green formulas (13a) and (13b) in $\Omega \backslash {\bar{\omega }}_{\varepsilon }$ and $\omega_{\varepsilon }$, respectively, we have for all $\phi \in H^{1}\left(\Omega \backslash \omega_{\varepsilon }\right)$: ϕ=0 on ∂Ω

$$\begin{array}{c} \int_{\Omega \backslash \omega_{\varepsilon }}\;\;\;{\left(\nabla \phi^{0}\right)}^{\;⊤}A^{0}\nabla \phi \;dx=-\int_{\Omega \backslash \omega_{\varepsilon }}\;\;\;\phi \;\mathrm{div}({\left(\nabla \phi^{0}\right)}^{\;⊤}A^{0})\;dx-\int_{\partial \omega_{\varepsilon }^{+}}{\left(\nabla \phi^{0}\right)}^{\;⊤}A^{0}\phi \nu \;dS_{x}, \end{array}$$

and for all $\phi \in H^{1}\left(\omega_{\varepsilon }\right)$:

$$\begin{array}{c} \int_{\omega_{\varepsilon }}\;\;\;{\left(\nabla \phi^{0}\right)}^{\;⊤}A^{0}\nabla \phi \;dx=-\int_{\omega_{\varepsilon }}\;\;\;\phi \;\mathrm{div}({\left(\nabla \phi^{0}\right)}^{\;⊤}A^{0})\;dx+\int_{\partial \omega_{\varepsilon }^{-}}{\left(\nabla \phi^{0}\right)}^{\;⊤}A^{0}\phi \nu \;dS_{x}. \end{array}$$

By summing these two expressions and by using the continuity of $\nabla \phi^{0}$ across the interface $\partial \omega_{\varepsilon }$, we insert the strong formulation (58) into the above right-hand sides and rewrite problem (56) in the disjoint domain $\Omega \backslash \partial \omega_{\varepsilon }$ as follows

(59) $$\begin{array}{cc} & \int_{\Omega \backslash \partial \omega_{\varepsilon }}({\left(\nabla \phi^{0}\right)}^{⊤}A^{0}\nabla \phi -\frac{\left|\Upsilon \backslash \omega \right|}{\left|\Upsilon \right|}\sum_{s=0}^{n}z_{s}e^{-\frac{z_{s}}{\kappa T}\phi^{0}}\phi )\;dx+\int_{\partial \omega_{\varepsilon }}{\left(\nabla \phi^{0}\right)}^{⊤}A^{0}\nu \left[\;\left[\phi \right]\;\right]\;dS_{x}=\int_{\Omega \backslash \partial \omega_{\varepsilon }}\frac{\left|\partial \omega \right|}{\left|\Upsilon \right|}g\phi \;dx\\ & \;\;\;\;\text{for all test-functions}\;\phi \in H^{1}\left(\Omega \backslash \partial \omega_{\varepsilon }\right):\phi =0\;\mathrm{on}\;\partial \Omega . \end{array}$$

In the following, we expand the terms in (59) based on the Lemmata 1–3. By applying the decomposition (51) of Lemma 3 to the integrand of the first term in the left-hand side of (59), we can represent it as the following sum

(60) $$\begin{array}{cc} {\left(\nabla \phi^{0}\right)}^{⊤}A^{0}\nabla \phi = & {\left(\nabla \phi^{0}\right)}^{⊤}(\left(\varepsilon DN^{\varepsilon }+I\right)A^{\varepsilon }-\varepsilon B^{\varepsilon })\nabla \phi \\ = & [(\nabla (\phi^{0}+\varepsilon {\left(\nabla \phi^{0}\right)}^{⊤}N^{\varepsilon }){)}^{\;⊤}A^{\varepsilon }-\varepsilon {\left(N^{\varepsilon }\right)}^{⊤}D\left(\nabla \phi^{0}\right)A^{\varepsilon }-{\left(\nabla \phi^{0}\right)}^{⊤}\varepsilon B^{\varepsilon }]\nabla \phi , \end{array}$$

where we have used that $[\nabla ({\left(\nabla \phi^{0}\right)}^{⊤}N^{\varepsilon }){]}^{⊤}={\left(\nabla \phi^{0}\right)}^{⊤}DN^{\varepsilon }+{\left(N^{\varepsilon }\right)}^{⊤}D\left(\nabla \phi^{0}\right).$

Next, the integral of the last function on the right-hand side of (60) can be integrated by parts by using (52) and (53) to calculate

(61) $$\begin{array}{cc} & -\int_{\Omega \backslash \partial \omega_{\varepsilon }}{\left(\nabla \phi^{0}\right)}^{⊤}\varepsilon B^{\varepsilon }\nabla \phi \;dx=\int_{\Omega \backslash \partial \omega_{\varepsilon }}\sum_{i,j,k=1}^{d}\phi_{,ij}^{0}\varepsilon b_{ijk,k}^{\varepsilon }\phi \;dx+\int_{\partial \omega_{\varepsilon }}\sum_{i,j,k=1}^{d}\phi_{,i}^{0}\varepsilon b_{ijk,k}^{\varepsilon }\nu_{j}\left[\;\left[\phi \right]\;\right]\;dS_{x}\\ & \;=-\int_{\Omega \backslash \partial \omega_{\varepsilon }}\sum_{i,j,k=1}^{d}\phi_{,ij}^{0}\varepsilon b_{ijk}^{\varepsilon }\phi_{,k}\;dx+\int_{\partial \omega_{\varepsilon }}({\left(\nabla \phi^{0}\right)}^{⊤}\varepsilon B^{\varepsilon }\nu -\sum_{i,j,k=1}^{d}\phi_{,ij}^{0}b_{ijk}^{\varepsilon }\nu_{k})\left[\;\left[\phi \right]\;\right]\;dS_{x}, \end{array}$$

with $b_{ijk}^{\varepsilon }\left(x\right):=b_{ijk}\left(\left\{\frac{x}{\varepsilon }\right\}\right)$. Substituting (60) and (61) in (59), we rewrite it

(62) $$\begin{array}{cc} & \int_{\Omega \backslash \partial \omega_{\varepsilon }}\;[(\nabla (\phi^{0}+\varepsilon {\left(\nabla \phi^{0}\right)}^{⊤}N^{\varepsilon }){)}^{\;⊤}A^{\varepsilon }\nabla \phi -\frac{\left|\Upsilon \backslash \omega \right|}{\left|\Upsilon \right|}\sum_{s=0}^{n}z_{s}e^{-\frac{z_{s}}{\kappa T}\phi^{0}}\phi ]\;dx+\int_{\partial \omega_{\varepsilon }}{\left(\nabla \phi^{0}\right)}^{⊤}\left(A^{0}+\varepsilon B^{\varepsilon }\right)\nu \left[\;\left[\phi \right]\;\right]\;dS_{x}\\ & \;=\int_{\Omega \backslash \partial \omega_{\varepsilon }}\frac{\left|\partial \omega \right|}{\left|\Upsilon \right|}g\phi \;dx+\varepsilon m_{\Omega \backslash \partial \omega_{\varepsilon }}(D\left(\nabla \phi^{0}\right),\nabla \phi )+m_{\partial \omega_{\varepsilon }}(D\left(\nabla \phi^{0}\right),\left[\;\left[\phi \right]\;\right]), \end{array}$$

where the bilinear continuous forms are given by

(63a) $$\begin{array}{cc} & m_{\Omega \backslash \partial \omega_{\varepsilon }}(D\left(\nabla \phi^{0}\right),\nabla \phi )\\ & \;:=\int_{\Omega \backslash \partial \omega_{\varepsilon }}({\left(N^{\varepsilon }\right)}^{⊤}D\left(\nabla \phi^{0}\right)A^{\varepsilon }\nabla \phi +\sum_{i,j,k=1}^{d}\phi_{,ij}^{0}b_{ijk}^{\varepsilon }\phi_{,k})\;dx, \end{array}$$

(63b) $$\begin{array}{cc} m_{\partial \omega_{\varepsilon }}(D\left(\nabla \phi^{0}\right),\left[\;\left[\phi \right]\;\right]) & :=\int_{\partial \omega_{\varepsilon }}\sum_{i,j,k=1}^{d}\phi_{,ij}^{0}b_{ijk}^{\varepsilon }\nu_{k}\left[\;\left[\phi \right]\;\right]\;dS_{x}. \end{array}$$

Next, we apply Lemma 2 with $f\left(x\right)=-\sum_{s=0}^{n}z_{s}\exp\left(-\frac{z_{s}}{\kappa T}\phi^{0}\left(x\right)\right)$ and obtain the following representation of the nonlinear term in (62)

(64) $$\begin{array}{cc} & -\frac{\left|\Upsilon \backslash \omega \right|}{\left|\Upsilon \right|}\sum_{s=0}^{n}\int_{\Omega \backslash \partial \omega_{\varepsilon }}z_{s}e^{-\frac{z_{s}}{\kappa T}\phi^{0}\left(x\right)}\phi \;dx\\ & \;\;=-\sum_{s=0}^{n}\int_{\Omega \backslash \omega_{\varepsilon }}z_{s}e^{-\frac{z_{s}}{\kappa T}\phi^{0}\left(x\right)}\phi \;dx+\varepsilon \;l_{2}\left(\phi \right). \end{array}$$

The boundary integral in (62) can be expanded by using (24) in Lemma 1, i.e.

$$\begin{array}{c} \int_{\Omega \backslash \partial \omega_{\varepsilon }}\frac{\left|\partial \omega \right|}{\left|\Upsilon \right|}g\phi \;dx=\int_{\partial \omega_{\varepsilon }^{-}}\varepsilon g\phi^{-}\;dS_{x}-\varepsilon \;l_{1}\left(\phi \right). \end{array}$$

Next, we subtract equation (62) for $\phi^{0}$ from the perturbed equation (15b) for $\phi^{\varepsilon }$ and use the notation $\phi^{1}:=\phi^{0}+\varepsilon {\left(\nabla \phi^{0}\right)}^{⊤}N^{\varepsilon }$. Moreover, for $\phi^{1}$, we remark that $[\;\left[\phi^{0}\right]\;]=0$ at $\partial \omega_{\varepsilon }$. Hence $\frac{\alpha }{\varepsilon }\left[\;\left[\phi^{1}\right]\;\right]=\alpha {\left(\nabla \phi^{0}\right)}^{⊤}\left[\;\left[N^{\varepsilon }\right]\;\right]={\left(\nabla \phi^{0}\right)}^{⊤}\left(A^{0}+\varepsilon B^{\varepsilon }\right)\nu$ in view of (54). Thus, after subtracting (62) from (15b), we calculate using the above relations

(65) $$\begin{array}{cc} & \int_{\Omega \backslash \partial \omega_{\varepsilon }}\;\;\;\nabla {\left(\phi^{\varepsilon }-\phi^{1}\right)}^{⊤}A^{\varepsilon }\nabla \phi \;dx+\int_{\partial \omega_{\varepsilon }}\frac{\alpha }{\varepsilon }\left[\;\left[\phi^{\varepsilon }-\phi^{1}\right]\;\right]\left[\;\left[\phi \right]\;\right]\;dS_{x}-\sum_{s=0}^{n}\int_{\Omega \backslash \omega_{\varepsilon }}\;\;\;z_{s}(e^{-\frac{z_{s}}{\kappa T}\phi^{\varepsilon }}-e^{-\frac{z_{s}}{\kappa T}\phi^{0}})\phi \;dx\\ & \;=\varepsilon \left(l_{1}\left(\phi \right)+l_{2}\left(\phi \right)\right)-\varepsilon \;m_{\Omega \backslash \partial \omega_{\varepsilon }}(D\left(\nabla \phi^{0}\right),\nabla \phi )-m_{\partial \omega_{\varepsilon }}(D\left(\nabla \phi^{0}\right),\left[\;\left[\phi \right]\;\right]). \end{array}$$

One difficulty is that $\phi^{1}$ cannot be substituted as test-function into (65) since $\phi^{1}\neq 0$ at the boundary ∂Ω. For its lifting, we take a cut-off function $\eta_{\varepsilon }$ supported in a ε-neighbourhood of ∂Ω such that $\eta_{\varepsilon }=1$ at ∂Ω. Hence, $\nabla \eta_{\varepsilon }=O\left(\frac{1}{\varepsilon }\right)$ and $|\mathrm{supp}\left(\eta_{\varepsilon }\right)|=O\left(\varepsilon \right)$ as $\varepsilon ↘0^{+}$. Due to the assumed ε-gap between ∂Ω and $\omega_{\varepsilon }$, we remark that $\mathrm{supp}(\eta_{\varepsilon })$ does not intersect $\partial \omega_{\varepsilon }$.

After substitution of $\phi =\phi^{\varepsilon }-\phi_{\eta_{\varepsilon }}^{1}$ with $\phi_{\eta_{\varepsilon }}^{1}:=\phi^{0}+\varepsilon \left(1-\eta_{\varepsilon }\right){\left(\nabla \phi^{0}\right)}^{⊤}N^{\varepsilon }$ into (65) and by using $\left[\;\left[\phi_{\eta_{\varepsilon }}^{1}\right]\;\right]=\left[\;\left[\phi^{1}\right]\;\right]$, we obtain the equality

(66) $$\begin{array}{cc} \int_{\Omega \backslash \partial \omega_{\varepsilon }} & \nabla {\left(\phi^{\varepsilon }-\phi^{1}\right)}^{⊤}A^{\varepsilon }\nabla \left(\phi^{\varepsilon }-\phi^{1}\right)\;dx+\int_{\partial \omega_{\varepsilon }}\frac{\alpha }{\varepsilon }{\left[\;\left[\phi^{\varepsilon }-\phi^{1}\right]\;\right]}^{2}\;dS_{x}\\ & -\sum_{s=0}^{n}\int_{\Omega \backslash \omega_{\varepsilon }}z_{s}(e^{-\frac{z_{s}}{\kappa T}\phi^{\varepsilon }}-e^{-\frac{z_{s}}{\kappa T}\phi_{\eta_{\varepsilon }}^{1}})\left(\phi^{\varepsilon }-\phi_{\eta_{\varepsilon }}^{1}\right)\;dx\\ = & -m_{\eta_{\varepsilon }}(\nabla \left(\phi^{\varepsilon }-\phi^{1}\right),D\left(\nabla \phi^{0}\right))-m_{\partial \omega_{\varepsilon }}(D\left(\nabla \phi^{0}\right),\left[\;\left[\phi^{\varepsilon }-\phi^{1}\right]\;\right])\\ & +\varepsilon \;\tilde{l}\left(\phi^{\varepsilon }-\phi_{\eta_{\varepsilon }}^{1}\right), \end{array}$$

where we introduce the form $m_{\eta_{\varepsilon }}$ due to the cut-off function as

(67) $$\begin{array}{cc} & m_{\eta_{\varepsilon }}(\nabla \left(\phi^{\varepsilon }-\phi^{1}\right),D\left(\nabla \phi^{0}\right))\\ & \;:=\varepsilon \int_{\mathrm{supp}(\eta_{\varepsilon })}\;\;\;\;\;\;\nabla {\left(\phi^{\varepsilon }-\phi^{1}\right)}^{⊤}A^{\varepsilon }\nabla (\eta_{\varepsilon }{\left(\nabla \phi^{0}\right)}^{⊤}N^{\varepsilon })\;dx, \end{array}$$

and the short notation $\tilde{l}$ stands for the following terms

(68) $$\begin{array}{c} \tilde{l}\left(\phi \right):=l_{1}\left(\phi \right)+l_{2}\left(\phi \right)-m_{\Omega \backslash \partial \omega_{\varepsilon }}(D\left(\nabla \phi^{0}\right),\nabla \phi )+m^{\varepsilon }\left(\phi^{0},\phi \right), \end{array}$$

where the nonlinear form $m^{\varepsilon }$ in (68) is given by

(69) $$\begin{array}{c} m^{\varepsilon }\left(\phi^{0},\phi \right):=\sum_{s=0}^{n}\int_{\Omega \backslash \omega_{\varepsilon }}\;\;\;z_{s}e^{-\frac{z_{s}}{\kappa T}\phi^{0}}\frac{1}{\varepsilon }(1-e^{-\varepsilon \left(1-\eta_{\varepsilon }\right)\frac{z_{s}}{\kappa T}{\left(\nabla \phi^{0}\right)}^{⊤}N^{\varepsilon }})\phi \;dx. \end{array}$$

From (69), it can be estimated uniformly as

(70) $$\begin{array}{c} |m^{\varepsilon }\left(\phi^{0},\phi \right)|\leq {K\|\phi \|}_{L^{2}\left(\Omega \backslash \omega_{\varepsilon }\right)}\leq K{\left\|\phi \right\|}_{H^{1}\left(\Omega \backslash \partial \omega_{\varepsilon }\right)},\;\left(K>0\right), \end{array}$$

due to the Taylor series $1-e^{-\varepsilon \xi }=\varepsilon \xi +o\left(\varepsilon \right)$ for small ε. To this end we remind that we have used the assumption of the higher regularity of the weak solutions $\phi^{0}$ and N. Therefore, $\nabla \phi^{0}$ and $N^{\varepsilon }$ are bounded uniformly, since $\phi^{0}$ does not depend on ε, and $N^{\varepsilon }$ by the homogeneity argument.

The left-hand side of (66) can be estimated from below by applying the coercivity of the matrix A as assumed in (6) and by observing that the third term on the left-hand side is nonnegative due to the strict monotonicity of the exponential function. Altogether with (21), this implies that

(71) $$\begin{array}{cc} K_{5}{\left\|\phi^{\varepsilon }-\phi^{1}\right\|}_{H^{1}\left(\Omega \backslash \partial \omega_{\varepsilon }\right)}^{2} & \leq |m_{\eta_{\varepsilon }}(D\left(\nabla \phi^{0}\right),\nabla \left(\phi^{\varepsilon }-\phi^{1}\right))|\\ & \;+|m_{\partial \omega_{\varepsilon }}(D\left(\nabla \phi^{0}\right),\left[\;\left[\phi^{\varepsilon }-\phi^{1}\right]\;\right])|+\varepsilon \left|\tilde{l}\left(\phi^{\varepsilon }-\phi_{\eta_{\varepsilon }}^{1}\right)\right|, \end{array}$$

with $K_{5}=K_{0}\left(\underline{K}+\alpha \right)>0$ after recalling $\underline{K}$ from (6) and $K_{0}$ from (21).

At this point, we remark that the right-hand side of (71) is a homogeneous function of degree one with respect to the norm $\|\phi^{\varepsilon }-\phi^{1}{\|}_{H^{1}\left(\Omega \backslash \partial \omega_{\varepsilon }\right)}$ as the following estimates will prove. Thus, the inequality (71) implies directly that the norm $\|\phi^{\varepsilon }-\phi^{1}{\|}_{H^{1}\left(\Omega \backslash \partial \omega_{\varepsilon }\right)}$ is bounded, which reconfirms estimate (20).

However, the following argument allows to refine the asymptotic residual estimate to obtain (57) as $\varepsilon ↘0^{+}$. In particular, we shall estimate above the three terms at the right-hand side of (71) and then apply Young’s inequality to obtain sums of sufficiently small terms of order $O(\|\phi^{\varepsilon }-\phi^{1}{\|}_{H^{1}\left(\Omega \backslash \partial \omega_{\varepsilon }\right)}^{2})$ and constant terms, which will constitute the refined residual estimate.

At first, from the estimates (25), (37), (70) and due to the boundedness of the bilinear form (63a) for $\phi \in H^{1}\left(\Omega \backslash \partial \omega_{\varepsilon }\right)$, it follows that

(72) $$\begin{array}{c} |\tilde{l}{\left(\phi \right)|\leq K\|\phi \|}_{H^{1}\left(\Omega \backslash \partial \omega_{\varepsilon }\right)},\;\left(K>0\right). \end{array}$$

Since $\phi_{\eta_{\varepsilon }}^{1}=\phi^{1}-\varepsilon \eta_{\varepsilon }{\left(\nabla \phi^{0}\right)}^{⊤}N^{\varepsilon }$ and recalling the properties of the cut-off function $\eta_{\varepsilon }$ implying $\int_{\mathrm{supp}(\eta_{\varepsilon })}{\left|\nabla \eta_{\varepsilon }\right|}^{2}\;dx=O\left(\frac{1}{\varepsilon }\right)$, we estimate that

(73) $$\begin{array}{c} \|\phi^{\varepsilon }-\phi_{\eta_{\varepsilon }}^{1}{\|}_{H^{1}\left(\Omega \backslash \partial \omega_{\varepsilon }\right)}^{2}\leq 2{\left\|\phi^{\varepsilon }-\phi^{1}\right\|}_{H^{1}\left(\Omega \backslash \partial \omega_{\varepsilon }\right)}^{2}+O\left(\varepsilon \right). \end{array}$$

Therefore, specifically for $\phi =\phi^{\varepsilon }-\phi_{\eta_{\varepsilon }}^{1}$, and by using Young’s inequality, it follows from (72) and (73) that

(74) $$\begin{array}{c} |\tilde{l}\left(\phi^{\varepsilon }-\phi_{\eta_{\varepsilon }}^{1}\right)|\leq K_{6}({\left\|\phi^{\varepsilon }-\phi^{1}\right\|}_{H^{1}\left(\Omega \backslash \partial \omega_{\varepsilon }\right)}^{2}+1),\;\left(K_{6}>0\right). \end{array}$$

For $\phi \in H^{1}\left(\Omega \backslash \partial \omega_{\varepsilon }\right)$, by using again Young’s inequality we estimate (67) with an arbitrary $t_{1}\in R_{+}$ by

(75) $$\begin{array}{c} |m_{\eta_{\varepsilon }}(\nabla \phi ,D\left(\nabla \phi^{0}\right))|\leq \varepsilon t_{1}K_{7}+\frac{1}{t_{1}}{\left\|\nabla \phi \right\|}_{L^{2}\left(\Omega \backslash \partial \omega_{\varepsilon }\right)}^{2},\;\left(K_{7}>0\right), \end{array}$$

and the form in (63b) by

(76) $$\begin{array}{c} |m_{\partial \omega_{\varepsilon }}(D\left(\nabla \phi^{0}\right),\left[\;\left[\phi \right]\;\right])|\leq \varepsilon t_{2}K_{8}+\frac{1}{\varepsilon t_{2}}{\left\|\left[\;\left[\phi \right]\;\right]\right\|}_{L^{2}\left(\partial \omega_{\varepsilon }\right)}^{2},\;\left(K_{8}>0\right), \end{array}$$

with an arbitrary $t_{2}\in R_{+}$. Therefore, by applying the estimates (74), (75) and (76) with $\phi =\phi^{\varepsilon }-\phi^{1}$ to (71) and for suitable $t_{1},t_{2}$ and $\varepsilon_{0}>0$ such that

$$0<K:=K_{5}-\left(\frac{1}{t_{1}}+\frac{1}{t_{2}}\right)K_{0}-\varepsilon_{0}K_{6},$$

we conclude

$$\begin{array}{c} K\|\phi^{\varepsilon }-\phi^{1}{\|}_{H^{1}\left(\Omega \backslash \partial \omega_{\varepsilon }\right)}^{2}\leq \varepsilon \left(t_{1}K_{7}+t_{2}K_{8}+K_{6}\right), \end{array}$$

for all $\varepsilon <\varepsilon_{0}$, which yields estimate (57). This finishes the proof. □

## 4. Discussion

In the following, we shall summarise the main observations concerning the presented results.

- We observe that the first two terms on the right-hand side of (71) express the residual error near ∂Ω and at $\partial \omega_{\varepsilon }$. These terms are asymptotically of order $O(\sqrt{\varepsilon })$ (as can be see by setting $t_{1}=O\left(\varepsilon^{-1/2}\right)=t_{2}$ in (75) and (76)) and thus constitute the leading order O(ε) in the residual error estimate (57). Therefore, by constructing corrector terms in form of the respective boundary layers, which is possible in practice for polyhedral domains with rational slopes of their flat boundaries with respect to the unit cell, the O(ε)-estimate (57) could be improved to the order $O(\varepsilon^{2})$.
- The factor $\frac{1}{\varepsilon }$ appears at the jump across interface $\partial \omega_{\varepsilon }$ in the left-hand side of microscopic equation (15b). It is controlled by the coercivity condition (21). In the homogenisation limit, this term disappears in the macroscopic equation (56). Instead it rather contributes to the macroscopic coefficients in (50).
- The factor ε in front of the inhomogeneous material parameter g, which is prescribed at the solid phase boundary $\partial \omega_{\varepsilon }^{-}$, presents the critical order. After averaging this factor guarantees the presence of the potential $\frac{\left|\partial \omega \right|}{\left|\Upsilon \right|}g$ distributed over the homogeneous domain Ω in (56).
- For variable functions $g(\left\{\frac{x}{\varepsilon }\right\})$ distributed periodically over the interface $\partial \omega_{\varepsilon }$, the decomposition
  $$\begin{array}{c} g={\left\langle g\right\rangle }_{y}+G,\;\;\text{with}\;{\left\langle g\right\rangle }_{y}:=\frac{1}{\left|\partial \omega \right|}\int_{\partial \omega }g\left(y\right)\;dy,\;{\left\langle G\right\rangle }_{y}=0, \end{array}$$
  yields in the limit $\varepsilon ↘0^{+}$ that the constant value ${\left\langle g\right\rangle }_{y}$ replaces g in the averaged problem (56), see e.g. [42].
- The nonlinear term appearing in (56) scales with the porosity coefficient $\frac{\left|\Upsilon \backslash \omega \right|}{\left|\Upsilon \right|}$.
